# Phase 1 and 2 Randomized Clinical Studies Determine Lack of Efficacy for Anti-IL-17C Antibody MOR106 in Moderate–Severe Atopic Dermatitis

**DOI:** 10.3390/jcm11237244

**Published:** 2022-12-06

**Authors:** Diamant Thaçi, Dave Singh, Mark Lee, Helen Timmis, Dominique Jacobs, Paul Passier, Susanne Rohrer, Johan Beetens, De Phung, Eric Sondag, Goran Babic, Guido Würth, Pia Kloepfer, Stefan Härtle, Silke Hüttner

**Affiliations:** 1 Institute and Comprehensive Center for Inflammation Medicine, University of Lübeck, 23538 Lübeck, Germany; 2 Medicines Evaluation Unit, Manchester University NHS Foundation Trust, University of Manchester, Manchester M23 9LT, UK; 3 Progressive Clinical Research, San Antonio, TX 78213, USA; 4 Galapagos, 2800 Mechelen, Belgium; 5 Novartis Pharma AG, 4056 Basel, Switzerland; 6 MorphoSys AG, 82152 Planegg, Germany

**Keywords:** atopic dermatitis, biological therapy, biologic, interleukin 17C, safety, efficacy, pharmacokinetics

## Abstract

Interleukin 17C (IL-17C) modulates epithelial inflammation and has a possible role in atopic dermatitis (AD) pathology. Four randomized clinical studies (Phase 1 and 2) investigated the safety, tolerability, efficacy, and pharmacokinetic profile of the anti-IL-17C monoclonal antibody MOR106 for up to 12 weeks (NCT03568071: *n* = 207 adults with moderate–severe AD; NCT03689829 Part 1: *n* = 32 healthy males; NCT03689829 Part 2: *n* = 44 adults with moderate–severe AD; and NCT03864627: *n* = 76 adults with moderate–severe AD). In these studies, MOR106 was either administered intravenously (i.v.) every 2 or 4 weeks at doses between 1–10 mg/kg, or subcutaneously (s.c.), either as a single dose or doses every 2 weeks at 320 mg. Overall, MOR106 was well-tolerated, and the safety profile was consistent with monoclonal antibodies approved for AD. Bioavailability following s.c. dosing was 55%, and steady-state drug levels were reached at 2–4 weeks. Ongoing studies were terminated following a futility analysis of the Phase 2 placebo-controlled dose-finding study (NCT03568071) due to a low probability for achieving the primary efficacy endpoint. Cumulatively, MOR106 demonstrated ineffectiveness for the treatment of AD, but its safety and pharmacokinetic characteristics warrant further drug development in other indications. Funding: sponsored by Galapagos NV; funded by Novartis AG.

## 1. Introduction

For information, a plain language summary that summarizes the manuscript is included in the Supplementary Materials.

The need for targeted treatments in atopic dermatitis (AD) and an increased understanding of AD pathophysiology is driving the development of new targeted treatments including biologics in AD [1,2]. Interleukin (IL)-17C is an epithelial-derived cytokine, thought to act via keratinocytes, epithelial cells, T helper type 2 and type 17 cells, and monocytes to amplify inflammation in the skin [3,4,5]. In vitro, IL-17C expression in keratinocytes is induced by other proinflammatory cytokines or bacteria, and IL-17C can induce expression of proinflammatory cytokines and antimicrobial peptides in keratinocytes [4,6,7]. In skin lesions of patients with AD, IL-17C messenger ribonucleic acid (mRNA) transcript expression is increased [8].

**Figure 1:**
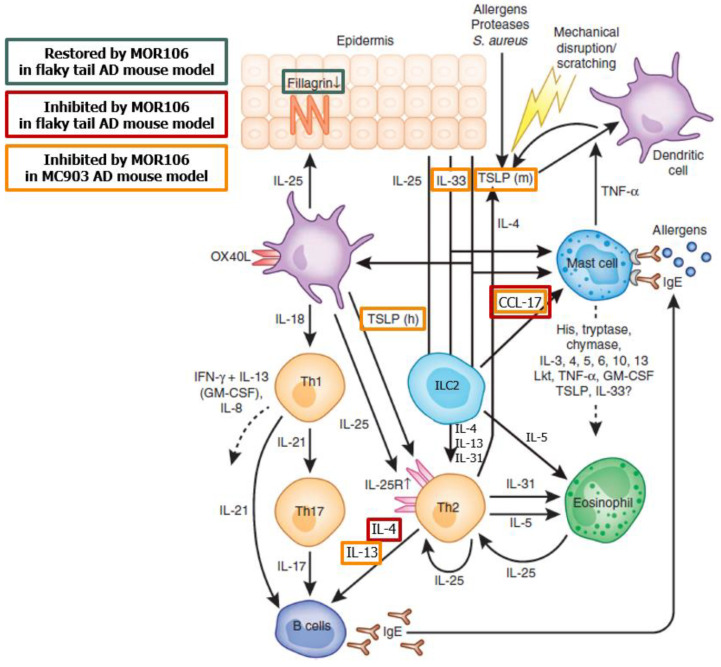
Molecular effects of MOR106 by neutralizing IL-17C in mouse models.

MOR106 is a human recombinant immunoglobulin (Ig) G1 monoclonal antibody that binds human IL-17C with high affinity to neutralize its biological activity [8]. In vitro, pro-inflammatory effects of IL-17C, including neutrophil recruitment, are reversed by MOR106 [4] and functional effects in vivo indicate IL-17C can mediate skin inflammation [8] (Figure 1 [8,9]). In the first in human, double-blind, randomized, placebo-controlled, dose-escalation study (NCT02739009) that evaluated single ascending doses in healthy individuals and multiple ascending doses in patients with moderate–severe AD, MOR106 was well-tolerated and improved eczema area and severity index (EASI) scores after four weekly doses and 10 weeks follow up [10].

Information based the potential mode of action of MOR106 in AD at the time the studies were conducted. Figure adapted from Volume 132, Cevikbas, F., et al. IL-33: a novel danger signal system in atopic dermatitis, pages 1326–1329, Copyright 2012 with permission from Elsevier [9]; for further information on effects of MOR106 in the flaky tail and MC903 models, see Vandeghinste, N. et al. [8]. 

CCL-17, CC chemokine ligand 17; IL, interleukin; ILC2, type 2 innate lymphoid cell; TNF-, Tumor Necrosis Factor alpha; and TSLP, thymic stromal lymphopoietin.

Here, we present the results of four Phase 1/Phase 2 clinical studies that further evaluated the safety and efficacy of MOR106 in healthy male study participants and patients with moderate–severe AD. A futility analysis of the Phase 2 intravenous (i.v.) dose-range finding study resulted in early termination of ongoing studies due to lack of efficacy. Consequently, the data presented are predominantly from the dose-range finding study.

## 2. Materials and Methods

### 2.1. Study Participants

Studies 1, 3 and 4 recruited males and females with: a diagnosis of chronic AD for ≥1 year (Hanifin and Rajka Criteria [11]); an EASI of ≥12 (studies 1 and 3) or ≥16 (study 4) at screening and ≥16 at baseline; an investigators’ global assessment (IGA) score ≥3 at screening and baseline; AD involvement of ≥10% body surface area at screening; and who were candidates for systemic therapy, aged 18–65 years, with a body mass index of 18–30 kg/m^2^ (studies 1 and 3) or ≤40 kg/m^2^ (study 4). Study 4 required ≥1 AD lesion, for which treatment with medium potency topical corticosteroids (TCS) was indicated.

Study 2 recruited healthy males aged 18–50 years. Full inclusion and exclusion criteria are presented in Supplementary Tables S1–S3.

### 2.2. Study Designs

**Figure 2:**
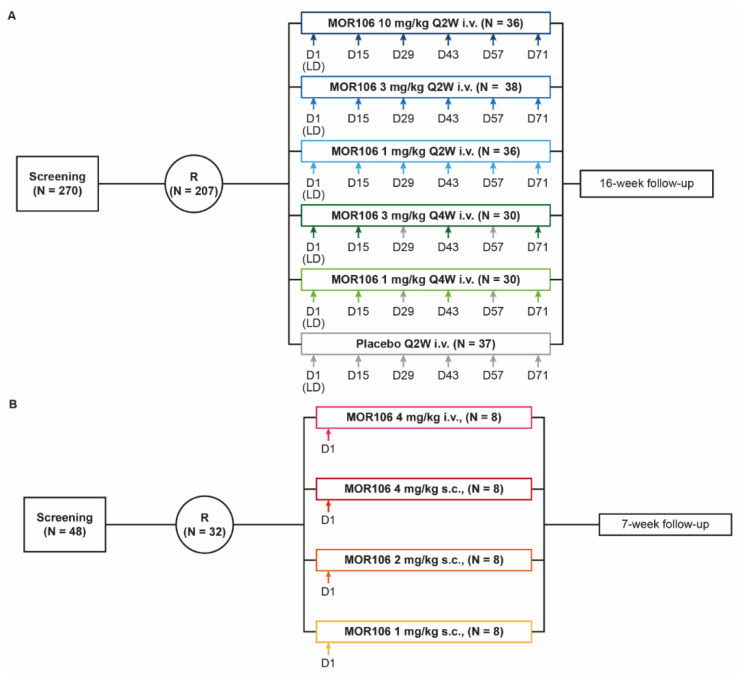

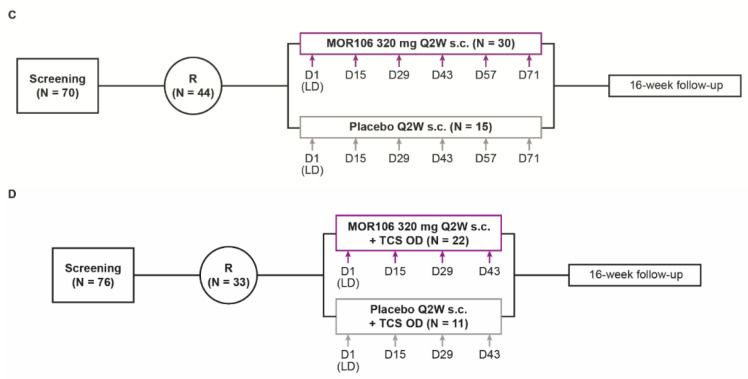
Study designs. (**A**) Study 1. Dose-range finding, multicenter, randomized, and placebo-controlled study: multiple dosing in adults with moderate–severe AD; (**B**) Study 2. Open-label, randomized study: single dose in healthy male study participants; (**C**) Study 3. Randomized, placebo-controlled study: multiple dosing in adults with moderate–severe AD; (**D**) Study 4. Multicenter, randomized, and placebo-controlled study: multiple dosing with concomitant topical corticosteroids in adult candidates for systemic therapy with moderate–severe AD. Arrows mark the dosing time points. TCS used according to the approved label. To maintain blinding in study 1, Q4W arms received alternating MOR106 and placebo. D: day; i.v.: intravenous; LD: loading dose (two times the regular dose); OD: once daily; Q2W: every 2 weeks; Q4W: every 4 weeks; R: randomization; s.c.: subcutaneous; TCS: topical corticosteroid.

Studies in patients with AD had a treatment period of 8–12 weeks, a 16-week follow up period (Figure 2A,C,D), and use of an emollient was required (≥2 times daily or, if applicable, once daily in areas of TCS application). In studies with multiple dosing, a loading dose (two times the regular dose) was administered on Day 1. Prespecified endpoints assessed efficacy, safety, tolerability and immunogenicity, and characterized the pharmacokinetic (PK) profile of MOR106 after i.v. or subcutaneous (s.c.) administration. Primary endpoints are described below, and secondary endpoints in the Supplementary Materials. Due to early termination, not all endpoints were assessed as planned (see ‘Statistical operations, Efficacy analyses’, Supplementary Materials). Due to the inclusion of other AD severity indices (EASI, IGA), Scoring Atopic Dermatitis data were considered redundant and not presented. Information on rescue therapy, concomitant medication, and statistical analyses are in the Supplementary Materials.

*Study 1* (*NCT03568071* [12]) Study 1 was a Phase 2, multicenter, randomized, and placebo-controlled dose-range finding study in adults with moderate–severe AD that evaluated repeated doses (MOR106 or placebo) administered every 2 weeks (Q2W) or every 4 weeks (Q4W) by i.v. infusion for 12 weeks. Patients were randomized (3:3:3:2:2:3) and stratified by country to either: MOR106 10 mg/kg Q2W, MOR106 3 mg/kg Q2W, MOR106 1 mg/kg Q2W, MOR106 3 mg/kg Q4W, MOR106 1 mg/kg Q4W, or placebo Q2W (Figure 2A). To maintain blinding, all patients received six infusions of 1 h (±10 min), administered Q2W; Q4W arms received alternating MOR106/placebo. The end of the treatment period was Day 85 (2 weeks after the last dose). The primary endpoint was % change in EASI score from baseline to Day 85.

*Studies 2 and 3* (*NCT03689829* [13]) NCT03689829 was a parallel-design Phase 1 study, conducted in two parts. Part 1 (study 2) was an open-label, randomized, and single-dose study (1, 2 or 4 mg/kg by s.c. injection, or 4 mg/kg by i.v. infusion) in healthy male study participants (Figure 2B). The primary endpoints were incidence of treatment-emergent adverse events (TEAEs (occurring following administration of MOR106, until withdrawal or end of follow up)), adverse events of special interest (AESIs (defined in ‘Safety Assessments’, Supplementary Materials)), serious adverse events (SAEs), and discontinuations due to adverse events (AEs) following administration of MOR106, PK parameters, area under the curve (AUC) ratio (4 mg/kg s.c. versus i.v. dosing), and dose proportionality for s.c. dosing. Part 2 (study 3) was a randomized, placebo-controlled study of multiple doses of MOR106 320 mg or placebo administered Q2W by s.c. injection for 12 weeks in adults with moderate–severe AD (Figure 2C). The primary endpoints were: incidence of TEAEs (end of treatment: Day 85 (2 weeks after the last dose)), AESIs, SAEs, and discontinuations due to AEs following administration of MOR106. Patients were randomized (2:1) to MOR106 320 mg (regular dose: 2 mL, 160 mg/mL) or placebo.

*Study 4* (*NCT03864627* [14]) Study 4 was a Phase 2, multicenter, randomized, and placebo-controlled study of multiple doses of MOR106 320 mg or placebo Q2W by s.c. injection with concomitant TCS for 8 weeks in adult candidates for systemic therapy with moderate–severe AD. Patients were randomized (2:1) to either MOR106 or placebo (Figure 2D). In addition to randomized treatment, all patients applied once-daily concomitant open-label and medium-potency TCS. Additional topical medications (lower-potency TCS and topical calcineurin inhibitors (TCI)) were permitted to treat AD lesions on the face, flexural, and genital areas but were limited to 2 days/week in areas where lesions cleared. During follow up, the topical treatment of residual active AD lesions continued at the investigator’s discretion. The primary endpoints were: incidence and severity of TEAEs (end of treatment: Day 57 (2 weeks after the last dose)), SAEs, and AESIs at either Day 169 or time of early discontinuation.

### 2.3. Safety and Efficacy Assessment

Details of efficacy (EASI, IGA, and pruritus numerical rating score (NRS)), safety, PK, pharmacodynamics (PD), immunogenicity assessments, and statistical operations are in the Supplementary Materials.

### 2.4. Ethics

All studies were conducted in accordance with the Declaration of Helsinki and its amendments and conformed with the protocol and International Council for Harmonization Guideline for Good Clinical Practice (ICH-GCP) [15,16]. An ‘Institutional Review Board Statement’ is included in the Supplementary Materials.

## 3. Results

### 3.1. Study Disposition and Demographics

Dates of enrolment, completion, and study participant disposition are shown in Supplementary Figure S1, baseline characteristics and demographics in Table 1 (patients with moderate–severe AD) and Supplementary Table S9 (healthy male study participants). In studies 1, 3, and 4: baseline EASI and IGA scores were indicative of moderate–severe AD, disease characteristics and demographics were balanced across treatment groups, mean age was 35.2, 32.9 and 37.4 years respectively, approximately half of patients were male, the majority were white, and mean body mass index was normal (except study 4, which was above ‘normal’ [17] weight). At the time of study termination, ~60% of patients in studies 1, 3, and 4 had received all doses of randomized treatment as planned (Supplementary Table S4).

### 3.2. PK, PD, and Immunogenicity

The MOR106 PK parameters established in healthy male study participants are summarized in Supplementary Table S10, and the MOR106 serum concentration profile over time in Supplementary Figure S2. The estimated relative bioavailability for MOR106 at 4 mg/kg s.c. (based on AUC_0–∞_) was 55% (90% confidence interval (CI): 48–63%). Dose-proportionality assessment across 1–4 mg/kg s.c. demonstrated an increase in maximum observed plasma concentration (C_max_) slightly greater than dose proportional (slope estimate: 1.27 (90% CI: 1.09–1.45)). AUC_0–t_ and AUC_0–∞_ increased in a dose-proportional manner.

**Figure 3:**
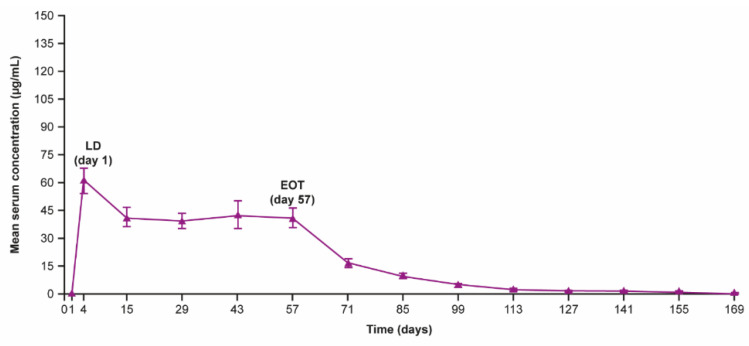
Mean MOR106 trough serum concentration over time obtained with MOR106 320 mg Q2W s.c. + TCS OD in study 4. Data are per the PK analysis set. Error bars show standard error of the mean. EOT: end of treatment; LD: loading dose (640 mg); OD: once daily; PK: pharmacokinetic; Q2W: every 2-weeks; TCS: topical corticosteroids.

MOR106 serum concentration following i.v. administration in study 1 and s.c. administration in studies 3 and 4 was in line with model-based predictions based on Phase 1 data. The loading dose led to steady state faster than would be expected based on the T_1/2_, resulting in steady-state drug levels within 2–4 weeks; a representative graph for study 4 is shown in Figure 3. 

Serum IL-17C was not detected prior to MOR106 dosing. After the start of MOR106 dosing, total (free and MOR106-bound) IL-17C serum concentration increased over 1–3 weeks, followed by approximately stable levels. Following MOR106 administration by i.v. infusion in study 1, absolute levels of total IL-17C ranged from ~1–3 ng/mL (depending on dose and dosing frequency), with an inter-individual variability between the minimum and maximum values at each time point of ~5- to 10-fold (Supplementary Figure S3); these results are representative of total IL-17C levels following MOR106 administration by s.c. injection in studies 3 and 4. 

Across all four studies, no antidrug antibodies (ADAs) were detected post dosing.

### 3.3. Efficacy

Efficacy data are presented in Table 2.

#### 3.3.1. Interim and Subsequent Futility Analysis of Study 1 Leading to Discontinuation of Ongoing Studies

A prespecified interim analysis of study 1 was conducted after approximately 90 patients completed treatment. This prompted an unplanned futility analysis that indicated a low probability for achieving a treatment difference of 40% versus placebo for the primary endpoint (% change in EASI score from baseline to Day 85). There were no concerns related to safety and tolerability. Ongoing studies of MOR106 in AD were discontinued, treatment stopped and, unless consent was withdrawn, patients entered the 16-week follow up.

#### 3.3.2. EASI

**Figure 4:**
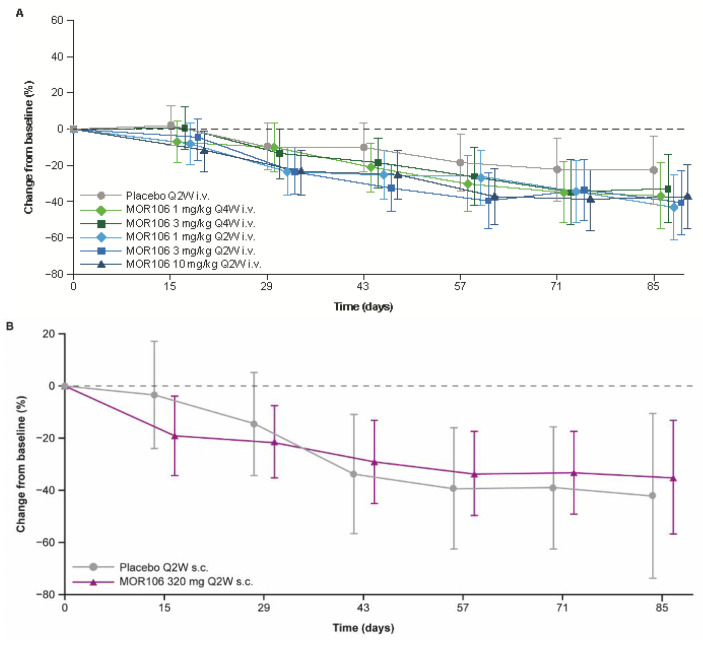

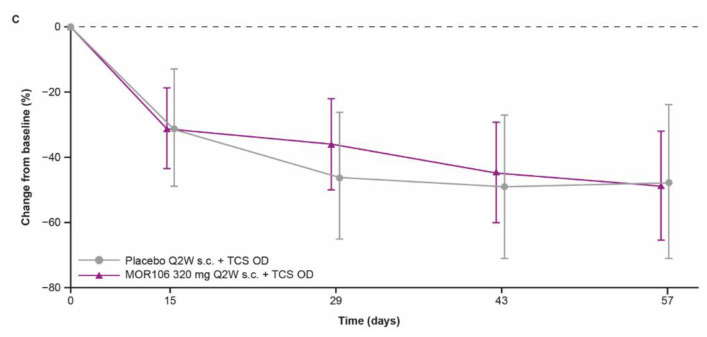
Percentage change in EASI score from baseline. (**A**) In study 1;(**B**) In study 3; (**C**) In study 4. Data are per full analysis set. Plots present least squares means; error bars show 95% confidence interval. A loading dose (two times the regular dose) was administered on Day 1. EASI: eczema area and severity index; i.v.: intravenous; Q2W: every 2 weeks; Q4W: every 4 weeks; s.c.: subcutaneous; TCS: topical corticosteroid.

In study 1, baseline EASI scores ranged between 16.0–72.0, and across treatment groups, the mean values were 28.5–34.3. The primary endpoint did not suggest a significant treatment effect with MOR106 (Figure 4A): the mean decrease in EASI score from baseline to Day 85 was 38.5% with placebo (EASI score: baseline, 29.2; Day 85, 15.9) and ranged between 46.6–57.9% in MOR106-treated groups (EASI score: baseline, 27.4–34.3; Day 85, 12.4–17.1 (depending on the treatment group, with no dose dependency apparent, see Figure 4A)). In the placebo group, the EASI score least squares (LS) mean % change from baseline at Day 85 was −22.1%; in MOR106-treated groups it ranged from −32.6% to −43.0% and was not significantly different from placebo (*p* > 0.05 for all comparisons, Table 2). The percentage of patients that achieved ≥50% improvement in EASI score from baseline (EASI50) in study 1, the odds ratio for EASI50 achievement and decrease in mean EASI score from baseline to end of follow up, were higher in MOR106-treated groups (Table 2). The changes in EASI score with MOR106 320 mg Q2W s.c. in study 3 (Figure 4B) and 4 (Figure 4C) did not suggest a treatment effect versus placebo.

#### 3.3.3. Investigators’ Global Assessment (IGA) Score

In study 1, the percentage of patients that achieved IGA of 0 or 1 (clear or almost clear) at Day 85 was 20% with placebo and ranged between 11.5–31.8% with MOR106 (depending on dose/dosing frequency, Table 2). In studies 3 and 4, IGA 0 or 1 was more often achieved with placebo than MOR106. 

#### 3.3.4. Pruritus

**Figure 5:**
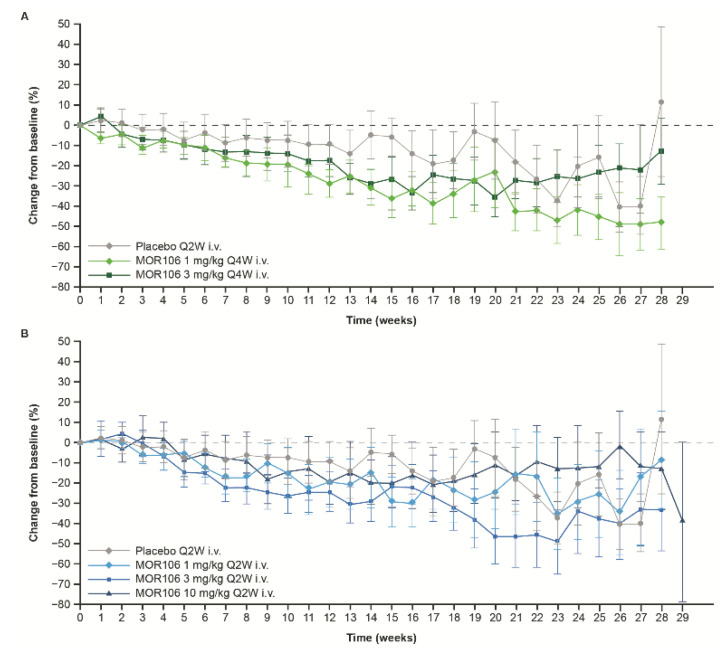

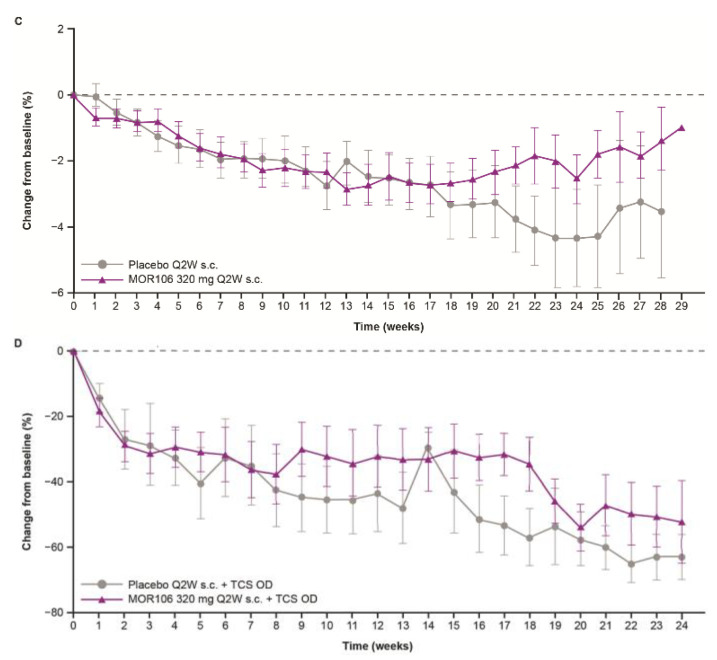
Percentage change in the pruritus numeric rating scale from baseline. (**A**) Study 1: placebo and MOR106 Q4W; (**B**) Study 1: placebo and MOR106 Q2W; (**C**) Study 3; (**D**) Study 4; Data are per full analysis set. Plots present mean values; error bars show standard error of the mean. A loading dose (two times the regular dose) was administered on Day 1. i.v.: intravenous; Q2W: every 2 weeks; Q4W: every 4 weeks; s.c.: subcutaneous; TCS: topical corticosteroid.

Pruritus NRS in study 1: the mean weekly % change in pruritus NRS from baseline at Week 12 was −9.7% with placebo and ranged between −17.7% and −29.1% in MOR106-treated groups (depending on dose/dosing frequency, Table 2). At most time points, and regardless of dose/dosing frequency, the standard error of the mean % change from baseline in MOR106 treatment groups overlapped with that of placebo (Figure 5A,B). Similarly, the weekly changes in pruritus NRS from baseline with MOR106 320 mg Q2W s.c. throughout study 3 (Figure 5C) and study 4 (Figure 5D) did not suggest a treatment effect versus placebo.

#### 3.3.5. Safety and Tolerability

No deaths were reported in any study.

#### 3.3.6. In Healthy Male Study Participants

TEAEs in study 2 are summarized in Supplementary Table S11 and presented by system organ class (SOC) and preferred term (PT) in Supplementary Table S12. The most frequently reported TEAEs in MOR106-exposed healthy male study participants were dermatitis acneiform (7/32 (21.9%)), rhinitis (5/32 (15.6%)), nasopharyngitis (3/32 (9.4%)) and headache (3/32 (9.4%)). The incidence of TEAEs with MOR106 1–4 mg/kg s.c. was independent of dose and similar with s.c. and i.v. administration (Supplementary Table S11).

#### 3.3.7. In Patients with Moderate–Severe AD

Treatment-emergent adverse events (TEAEs) in studies 1, 3, and 4 are summarized in Table 3; TEAEs by SOC and PT with an overall incidence ≥5% in Studies 1, 3, and 4 are presented in Supplementary Tables S5–S7, respectively; and skin-related TEAEs by SOC and PT in studies 1, 3, and 4 are presented in Supplementary Table S8. Overall, MOR106 by i.v. and s.c. administration was well-tolerated. The incidence of TEAEs was well-balanced between groups in studies 1, 3, and 4, except with the MOR106 1 mg/kg Q4W group in study 1, where TEAEs were ~20% more frequent than placebo.

In study 1, the SOCs with the highest incidence of TEAEs were ‘skin and subcutaneous tissue disorders’, ‘infections and infestations’, and ‘nervous system disorders’, predominantly due to worsening of AD, nasopharyngitis, upper respiratory tract infection, and headache. In general, TEAEs were well-balanced between treatment groups, with no dose relationship apparent with MOR106. TEAEs leading to permanent discontinuation occurred in 8.1% of the placebo group and between 2.6–13.3% of MOR106-treated patients. The most common reason for discontinuation was exacerbation of AD.

The safety profiles in studies 3 and 4 were broadly in line with study 1, except a minor imbalance in TEAE frequency in study 3 (more TEAEs overall, and skin- and drug-related TEAEs with MOR106 320 mg Q2W s.c. versus placebo). Data from study 4 did not suggest that concomitant TCS administration affected safety outcomes. 

Rates of AESI were low. In study 1, skin-related AESIs were reported in 24 patients and were more frequent with MOR106 than placebo (placebo: 2.7%; MOR106, 2.8–22.2%, with the highest frequency in the MOR106 10 mg/kg Q2W group). Few injection site reactions (ISRs) of grade ≥ 2 occurred.

In studies 1–3, most TEAEs were of moderate intensity; in study 4, the majority were mild. In study 1, a total of 17 serious TEAEs occurred (5.4% of placebo-treated patients and between 2.6–16.7% of MOR106-treated patients), depending on dose/dosing frequency. The majority (9/17) were exacerbations of AD, with no apparent differences between groups. One patient treated with MOR106 1 mg/kg Q4W s.c. experienced a life-threatening SAE: appendicitis with consecutive surgery, deemed unrelated to MOR106 by the investigator. In studies 3 and 4, a total of two and one SAEs occurred in MOR106-treated patients, respectively, and none with placebo. 

There were no apparent trends in electrocardiogram, vital signs, or treatment-emergent laboratory abnormalities; abnormalities in physical examination were dominated by skin-related AEs.

## 4. Discussion

The four studies established the MOR106 PK profile and demonstrated good bioavailability with MOR106 320 mg Q2W s.c. dosing in patients with moderate–severe AD. The use of a loading dose resulted in steady-state drug levels within 2–4 weeks. The futility analysis of EASI score in study 1 indicated a low probability of achieving the 40% treatment difference versus placebo specified by the primary efficacy endpoint, and resulted in early termination of ongoing MOR106 studies in AD. Subsequent data analyses confirmed a lack of efficacy for MOR106 in moderate–severe AD. 

The binding of MOR106 to IL-17C increases the terminal elimination half-life of the bound cytokine, and consequently total serum IL-17C increases from undetectable levels until a steady state is reached. Thus, the target binding of IL-17C by MOR106 was indirectly confirmed by an increase in total serum IL17-C after the start of treatment (observed after 2–3 weeks).

In terms of efficacy, changes in EASI and IGA scores and the pruritus NRS consistently indicated an absence of a treatment effect and, importantly, no dose response. 

Folliculitis and acne observed in some healthy male study participants in study 2 suggested that biological activity of unclear nature occurred in the dermal epithelium with MOR106 but, overall, the data from all studies indicated targeted therapy for up to 12 weeks to neutralize IL-17C signaling has no impact on pathology in moderate–severe AD.

Responses to placebo in studies 3 and 4 were sizeable, with >40% reduction in the LS mean EASI score with placebo at the end of treatment in both studies. Considerable placebo responses have been noted in clinical trials of lebrikizumab and tralokinumab in AD, potentially due to effects of concomitant TCS, which may have contributed to the placebo response in study 4 [18,19].

The MOR106 safety profile was in line with monoclonal antibodies approved in AD (dupilumab [20,21,22] and tralokinumab [23,24,25]). MOR106 was well-tolerated following i.v. and s.c. administration, and the rates of ISRs were comparable to placebo. Elevated rates of conjunctivitis that have been observed in dupilumab AD trials did not occur with MOR106 [20]. No ADA formation with MOR106 was detected, indicating absence of immunogenicity. In patients with moderate–severe AD, the most common TEAEs were skin disorders related to AD exacerbation, and the most frequent skin-related AESI were folliculitis and acne. Interestingly, following MOR106 administration, acneiform eruptions were also observed in >20% of the healthy males who participated in Study 2, suggesting biological activity of unclear nature in the skin epithelium. Overall, the data from the four studies demonstrated no treatment effect in moderate–severe AD.

The studies had several limitations. In all studies, the majority of patients were white, and the relevance of these data to other racial groups is uncertain. Early termination of the MOR106 clinical program in AD resulted in the inclusion of fewer patients than planned in data sets used for efficacy analyses. In line with many Phase 2 trials, no corrections for multiplicity were performed.

## 5. Conclusions

MOR106 administered by i.v. infusion or s.c. injection was well-tolerated in moderate–severe AD, with no immunogenicity detected following MOR106 administration. The PK profile of MOR106 was established and demonstrated good bioavailability with MOR106 320 mg Q2W s.c. dosing in patients with moderate–severe AD. MOR106 doses of up to 10 mg/kg Q2W administered i.v. and up to 320 mg Q2W administered s.c. demonstrated a lack of efficacy for the treatment of moderate–severe AD. Although the IL-17C axis is an interesting target, it is unlikely to be an effective target for the treatment of AD.

## Figures and Tables

**Table 1:** Baseline demographics and characteristics, studies in moderate–severe AD.

	Study 3 (MOR106/Placebo Administered by s.c. Injection)							Study 3 (MOR106/Placebo Administered by s.c. Injection)			Study 4 (MOR106/Placebo Administered by s.c. Injection)		
	Placebo Q2W (*n* = 37)	MOR106 1 mg/kg Q2W (*n* = 36)	MOR106 3 mg/kg Q2W (*n* = 38)	MOR106 10 mg/kg Q2W (*n* = 36)	MOR106 1 mg/kg Q4W (*n* = 30)	MOR106 3 mg/kg Q4W (*n* = 30)	Total (*n* = 207)	Placebo Q2W (*n* = 14)	MOR106 320 mg Q2W (*n* = 29)	Total (*n* = 43)	Placebo Q2W + TCS OD (*n* = 11)	MOR106 320 mg Q2W + TCS OD (*n* = 22)	Total (*n* = 33)
Age, years	38.1	35.1	35·0	33.8	35.6	33.7	35.2	32.4	33.1	32.9	39.8	36.1	37.4
Mean (range)	(18–65)	(18–64)	(18–65)	(18–62)	(20–64)	(18–64)	(18–65)	(18–48)	(19–63)	(18–63)	(21–64)	(18–54)	(18–64)
Male, *n* (%)	19 (51.4)	16 (44.4)	19 (50.0)	26 (72.2)	13 (43.3)	13 (43.3)	106 (51.2)	10 (71.4)	16 (55.2)	26 (60.5)	4 (36.4)	11 (50.0)	15 (45.5)
Race, *n* (%)													
Asian	0	0	0	0	1 (3.3)	0	1 (0.5)	1 (7.1)	4 (13.8)	5 (11.6)	1 (9.1)	5 (22.7)	6 (18.2)
Black/African American	NR	NR	NR	NR	NR	NR	NR	0	1 (3.4)	1 (2.3)	1 (9.1)	2 (9.1)	3 (9.1)
White	37 (100)	36 (100)	38 (100)	36 (100)	29 (96.7)	30 (100)	206 (99.5)	13 (92.9)	24 (82.8)	37 (86.0)	9 (81.1)	15 (68.2)	24 (72.7)
Weight, kg	74.9	71.7	72.7	75.6	73.7	72.9	73.6	74.2	74.2	74.2	81.3	74	76.4
Mean (range)	(48.8–102.0)	(44.2–107.2)	(48.8–100.5)	(47.0–107.5)	(47.4–102.4)	(51.2–121.0)	(44.2–121.0)	(61.6–88.0)	(49.2–114.5)	(49.2–114.5)	(59.8–121.0)	(54.0–99.0)	(54.0–121.0)
BMI, kg/m^2^	25.2	24.1	25	25.1	24.8	24.2	24.8	23.6	25	24.6	29.8	26.9	27.9
Mean (range)	(19.2–29.7)	(18.1–29.7)	(18.4–29.6)	(19.1–29.8)	(18.3–29.6)	(18.5–33.9)	(18.1–33.9)	(20.1–27.3)	(18.7–31.1)	(18.7–31.1)	(22.0–37.8)	(18.0–36.5)	(18.0–37.8)
EASI score	29.2	29	29	34.3	28.5	27.4	29.6	22.06	24.16	23.46	21	25.9	24.2
Mean (range)	(16.0–66.4)	(16.1–49.2)	(16.2–72.0)	(16.4–72.0)	(16.0–64.8)	(16.2–49.2)	(16.0–72.0)	(16.1–37.2)	(16.0–53.0)	(16.0–53.0)	(16.2–30.0)	(16.2–53.3)	(16.2–53.3)
IGA score													
3, *n* (%)	20 (54.1)	24 (66.7)	24 (63.2)	24 (63.2)	17 (56.7)	22 (73.3)	131 (63.3)	–	–	–	10 (90.9)	14 (63.6)	24 (72.7)
4, *n* (%)	17 (45.9)	12 (33.3)	14 (36.8)	12 (33.3)	13 (43.3)	8 (26.7)	76 (36.7)	–	–	–	1 (9.1)	8 (36.4)	9 (27.3)
Mean (range)	–	–	–	–	–	–	–	3.1 (3–4)	3.4 (3–4)	3.3 (3–4)	–	–	–
Use non-biologic systemic drugs, *n* (%)	30 (81.1)	25 (69.4)	24 (63.2)	25 (69.4)	24 (80.0)	21 (70.0)	149 (72.0)	10 (71.4)	18 (64.3)	28 (66.7)	4 (36.4)	8 (36.4)	12 (36.4)
Use phototherapy, * *n* (%)	10 (27.0)	11 (30.6)	11 (28.9)	10 (28.6)	13 (43.3)	9 (30.0)	64 (31.1)	7 (50.0)	9 (33.3)	16 (39.0)	1 (9.1)	1 (4.5)	2 (6.1)

* Information missing for one patient in the MOR106 10 mg/kg Q2W group. All studies: data are per the full analysis set; a loading dose (two times the routine dose) was administered on Day 1. AD: atopic dermatitis; EASI: eczema area and severity index; BMI: body mass index; IGA: investigators’ global assessment; i.v.: intravenous; NR: not recruited; OD: once daily; Q2W: every 2 weeks; Q4W: every 4 weeks; s.c.: subcutaneous; TCS: topical corticosteroid.

**Table 2:** Summary of efficacy data, studies in moderate–severe AD.

	Study 1 (MOR106/Placebo Administered by i.v. Infusion)						Study 3 (MOR106/Placebo Administered by s.c. Injection)		Study 4 (MOR106/Placebo Administered by s.c. Injection)	
	Placebo Q2W	MOR106 1 mg/kg Q2W	MOR106 3 mg/kg Q2W	MOR106 10 mg/kg Q2W	MOR106 1 mg/kg Q4W	MOR106 3 mg/kg Q4W	Placebo Q2W	MOR106 320 mg Q2W	Placebo Q2W + TCS OD	MOR106 320 mg Q2W + TCS OD
EASI score, mean (median) [range]										
Baseline	*n* = 37 29.2 (26.1) [16.0–66.4]	*n* = 36 29.0 (28.1) [16.1–57.5]	*n* = 38 29.0 (27.0) [16.2–72.0]	*n* = 36 34.3 (27.8) [16.24–72.0]	*n* = 30 28.5 (26.2) [16.0–64.8]	*n* = 30 27.4 (25.7) [16.2–49.2]	*n* = 14 22.1 (18.3) [16.1–37.2]	*n* = 28 24.2 (21.8) [16.0–53.0]	*n* = 11 21.0 (19.3) [16.2–30.0]	*n* = 22 25.9 (21.9) [16.2–53.3]
Day 57 or 85 *	*n* = 20 15.9 (18.3) [0.6–41.2]	*n* = 25 12.6 (11.0) [0.2–45.7]	*n* = 24 14.2 (9.1) [0.0–49.7]	*n* = 26 17.1 (13.3) [0.0–49.7]	*n* = 25 14.4 (13.8) [0.4–56.2]	*n* = 22 12.4 (8.6) [0.0–54.0]	*n* = 9 9.1 (5.9) [2.0–19.6]	*n* = 20 15.7 (13.0) [1.8–63.6]	*n* = 8 9.8 (9.7) [1.2–22.4]	*n* = 16 10.9 (10.7) [0.0–21.2]
Day 169 or 197 ^†^	*n* = 9 10.9 (9.6) [1.0–25.0]	*n* = 9 7.7 (5.6) [1.0–23.0]	*n* = 9 7.4 (2.8) [0.0–21.6]	*n* = 18 14.0 (8.1) [0.0–60.0]	*n* = 7 9.4 (3.2) [0.4–40.0]	*n* = 11 8.1 (6.4) [0.4–20.2]	*n* = 4 2.9 (2.4) [1.2–5.6]	*n* = 11 12.9 (10.0) [0.0–32.6]	*n* = 6 6.9 (2.8) [0.6–26.4]	*n* = 9 10.3 (8.8) [2.4–21.4]
EASI score % change from baseline at Day 85 or 57 *										
LS mean (SE)	–22.1 (9.45)	−43.0 (8.94)	−40.2 (9.13)	−37.0 (8.90)	−36.2 (9.07)	–32.6 (9.49)	−42.2 (15.54)	−35.0 (10.66)	−46.8 (11.45)	−48.8 (8.06)
LS mean difference vs. placebo at Day 57 or 85, * (95% CI), *p*-value	n/a	−20.9 (−44.6; 2.9), *p* = 0.08	−18.1 (−42.1; 6.0), *p* = 0.14	−14.9 (−38.7; 8.9), *p* = 0.22	−14.2 (−38.3; 10.0), *p* = 0.25	−10.5 (−35.2; 14.2), *p* = 0.40	n/a	−7.2 (30.8; 45.2), not tested	n/a	2.0 (31.4; 27.4), not tested
% achieving EASI50, odds ratio (95% CI)	40.0, 1.50 (0.48; 4.74)	64.0, 2.13 (0.72; 6.34)	66.7, 2.30 (0.70; 7.51)	61.5, 2.23 (0.72; 6.90)	48.0, 1.50 (0.48; 4.74)	63.6, 2.11 (0.66; 6.71)	n/a	n/a	n/a	n/a
% achieved IGA score of 0 or 1 at Day 57 or 85 *	20.0	12.0	20.8	11.5	20.0	31.8	33.3	5.0	25.0	18.8
Pruritus NRS, mean weekly % change from baseline in the last week of treatment ^‡^	−9.7	−19.7	−24.5	−20.0	−29.1	−17.7	−41.7	−35.3	−37.3	−32.9

All studies: data are per the full analysis set. * At Day 85 for studies 1 and 3 and Day 57 for study 4. ^†^ At Day 197 for studies 1 and 3 and Day 169 for study 4. **^‡^** Mean weekly % change from baseline at Week 12 (study 1 and 3) or at Week 8 (study 4). AD: atopic dermatitis; EASI: eczema area and severity index; EASI50: ≥50% reduction in EASI score from baseline; IGA: investigators’ global assessment; i.v.: intravenous; LS: least squares; n/a: not applicable; NRS: numerical rating score; OD: once daily; Q2W: every 2 weeks; Q4W: every 4 weeks; s.c.: subcutaneous; SE: standard error of the mean; TCS: topical corticosteroid.

**Table 3:** Treatment-emergent adverse events, patients with moderate–severe AD.

Data are *n* (%)	Study 1 (MOR106/Placebo Administered by i.v. Infusion)						Study 3 (MOR106/Placebo Administered by s.c. Injection)		Study 4 (MOR106/Placebo Administered by s.c. Injection)	
	Placebo Q2W (*n* = 37)	MOR106 1 mg/kg Q2W (*n* = 36)	MOR106 3 mg/kg Q2W (*n* = 38)	MOR106 10 mg/kg Q2W (*n* = 36)	MOR106 1 mg/kg Q4W (*n* = 30)	MOR106 3 mg/kg Q4W (*n* = 30)	Placebo Q2W (*n* = 14)	MOR106 320 mg Q2W (*n* = 29)	Placebo Q2W + TCS OD (*n* = 11)	MOR106 320 mg Q2W + TCS OD (*n* = 22)
TEAE	26 (70.3)	22 (61.1)	29 (76.3)	26 (72.2)	27 (90.0)	22 (73.3)	11 (78.6)	25 (86.2)	5 (45.5)	11 (50.0)
Serious TEAE	2 (5.4)	4 (11.1)	1 (2.6)	2 (5.6)	5 (16.7)	3 (10.0)	0	2 (6.9)	0	1 (4.5)
Infusion related or injection site reaction *	0	1 (2.8)	0	1 (2.8)	1 (3.3)	0	0	1 (3.4)	1 (9.1)	3 (13.6)
Skin-related TEAE	1 (2.7)	1 (2.8)	7 (18.4)	8 (22.2)	4 (13.3)	3 (10.0)	3 (21.4)	9 (31.0)	2 (18.2)	1 (4.5)
Worst TEAE intensity:										
Mild	7 (18.9)	5 (13.9)	8 (21.1)	7 (19.4)	7 (23.3)	5 (16.7)	2 (14.3)	3 (10.3)	4 (36.4)	8 (36.4)
Moderate	16 (43.2)	12 (33.3)	17 (44.7)	18 (50.0)	13 (43.3)	13 (43.3)	8 (57.1)	19 (65.5)	1 (9.1)	2 (9.1)
Severe	3 (8.1)	5 (13.9)	4 (10.5)	1 (2.8)	6 (20.0)	4 (13.3)	1 (7.1)	3 (10.3)	0	1 (4.5)
Life threatening	0	0	0	0	1 (3.3)	0	0	0	0	0
Drug-related TEAE	6 (16.2)	8 (22.2)	11 (28.9)	12 (33.3)	6 (20.0)	8 (36.7)	4 (28.6)	13 (44.8)	3 (27.3)	7 (31.8)
Study treatment:										
Temporarily stopped	0	1 (2.8)	1 (2.6)	0	0	0	0	1 (3.4)	0	0
Permanently stopped	3 (8.1)	4 (11.1)	1 (2.6)	1 (2.8)	3 (10.0)	4 (13.3)	2 (14.3)	3 (10.3)	0	1 (4.5)

Data are *n* (%) for safety population analysis set, from start of treatment to follow up visit. * Depending on method of study drug administration. AD: atopic dermatitis; i.v.: intravenous; OD: once daily; Q2W: every 2 weeks; Q4W: every 4 weeks; s.c.: subcutaneous; TCS: topical corticosteroid; TEAE: treatment-emergent adverse event.

## Data Availability

Individual participant data are not publicly available.

## Supplementary Materials

The following supporting information can be downloaded at: https://www.mdpi.com/article/10.3390/jcm11237244/s1, Plain language summary. Additional methods: Inclusion criteria: Table S1: Full inclusion criteria, studies 1–4. Exclusion criteria: Table S2: Full exclusion criteria, studies 1–4. Table S3: List of prohibited medicines and therapies. Secondary endpoints. Settings and locations. Assigning individuals to treatment groups. Blinding and unblinding. Concomitant medications. Efficacy assessments. Safety assessments. PK, PD, and immunogenicity assessments. Statistical operations. Efficacy analyses. Rescue medication. Handling of missing data. Sample size determination. Statistical model for relative bioavailability and dose proportionality. Additional results: Study participant disposition and dates of study conduct. Figure S1: Study participant disposition and dates of study conduct, studies 1–4. Adherence to planned dosing schedules. Patients with moderate–severe AD: Table S4: Number of infusions/injections received, patients with moderate–severe AD. Healthy male study participants (Study 2). Additional safety data, patients with moderate–severe AD. Table S5: TEAEs by SOC and PT with overall incidence ≥5% in patients with moderate–severe AD, study 1. Table S6: TEAEs by SOC and PT with overall incidence ≥5% in patients with moderate–severe AD, study 3. Table S7: TEAEs by SOC and PT with overall incidence ≥5% in patients with moderate–severe AD, study 4. Table S8: Skin-related TEAEs by SOC and PT in patients with moderate–severe AD, studies 1, 3, and 4. Baseline demographics and characteristics, healthy male study participants. Table S9: Summary of baseline demographics and characteristics, healthy male study participants. PK profile, healthy male study participants. Figure S2: Mean MOR106 serum concentration over time with i.v. and s.c. administration of a single dose of MOR106 in healthy male study participants. Table S10: Summary of MOR106 PK parameters, healthy male study participants. Target binding of IL-17C by MOR106 in patients with moderate–severe AD. Figure S3: Serum concentration of total (free and MOR106-bound) IL-17C in study 1. Safety, healthy male study participants. Table S11: Summary of TEAEs in healthy male study participants. Table S12: Summary of TEAEs by SOC and PT in healthy male study participants occurring in ≥2 individuals. Table S13: Ethics committee approval information. References [26,27] are cited in Supplementary Materials.
