# Supplementary material for: Phase 1 and 2 Randomized Clinical Studies Determine Lack of Efficacy for Anti-IL-17C Antibody MOR106 in Moderate–Severe Atopic Dermatitis
Source: J Clin Med. 2022 Dec 6;11(23):7244. doi: 10.3390/jcm11237244 (PMC9740200; doi:10.3390/jcm11237244)
Supplement: Supplementary file 1 [file jcm-11-07244-s001.zip › jcm-1971659-supplementary.pdf]

Supplementary Materials

Plain language summary

Atopic dermatitis (AD) – sometimes referred to as “eczema” – is a condition that causes skin to become red and itchy due to inflammation from a dysregulated immune system and impairment of the skin barrier. AD has a profoundly negative impact on quality of life and new, more effective systemic treatments are being sought; especially for more severe cases that cannot be managed with topical treatment (i.e. treatment applied to the skin) alone.

Interleukin-17C (IL-17C) is a messenger protein that, when activated, triggers the immune system to respond to infection. Experiments have shown that the presence of IL-17C can lead to inflammation in the skin and suggest that it has a causative role in AD. MOR106 is a drug that binds to and neutralizes the activity of IL-17C and therefore may be of benefit in AD.

We report the outcomes from four clinical studies (some in healthy males; some in patients with moderate to severe AD) that were conducted to determine:

- how rapidly MOR106 is absorbed into the circulation;
- what dose of MOR106 is required to achieve an appropriate level in the circulation;
- how the administration method (intravenous: administered through a drip into a vein in the arm; subcutaneous injection: injected under the skin in a region around the stomach) might affect MOR106 levels in the circulation;
- whether MOR106 binds IL-17C in the circulation as expected;

- the safety (side effect) profile of the drug in people;
- whether MOR106 has any benefit regarding the signs and symptoms of AD.

Our data established the dosing required to achieve an appropriate and stable level of MOR106 when administered by intravenous or subcutaneous injection; this was achieved with the doses tested, following dosing every 2 or 4 weeks. Our data showed that after MOR106 was administered it bound IL-17C in the bloodstream as expected; however, across all four studies it was clear that MOR106 did not markedly improve the signs and symptoms of AD compared with placebo. In one study of 207 adults with moderate to severe AD, an early analysis of the data was conducted and found that MOR106 did not achieve a key measure of AD symptom-improvement. At this point, the decision was made to halt all ongoing studies, as it would have been unethical to continue without a tangible benefit for the patients involved. No serious safety concerns were raised. The patients in the studies experienced side effects that were in line with biologic treatments already approved for AD. Overall, our data show MOR106 is not an effective treatment for the signs and symptoms of moderate to severe AD.

Additional methods

Inclusion criteria

Supplementary Table S1: Full inclusion criteria, studies 1–4

<table>
<tr><th colspan="5">Inclusion criteria</th></tr>
<tr><td colspan="5">All studies, unless otherwise stated:
• Able and willing to give voluntary, written informed consent and to agree to the schedule of assessments.
• Met all inclusion criteria and no exclusion criteria.
• Willing to adhere to the following three contraceptive restrictions:
1. Female study participants of childbearing potential must have a negative serum pregnancy test at screening, and a negative urine pregnancy test at baseline (not applicable to study 2)
2. Female study participants of childbearing potential must use a highly effective method of contraception from 28 days prior to the first dose of study medication, during the study, and for ≥24 weeks after the last dose of study medication (not applicable to study 2)
3. Non-vasectomized male study participants with a female partner of childbearing potential must agree to use a condom in addition to another highly effective form of contraception from prior to the first dose, during the study, and for ≥24 weeks after last dose of study medication (for study 1, use of a condom was not required until administration of the first dose of study medication).</td></tr>
<tr><td></td><td>Study 1
(NCT03568071[1])</td><td>Study 2
(NCT03689829[2])</td><td>Study 3
(NCT03689829[2])</td><td>Study 4
(NCT03864627[3])</td></tr>
<tr><td>Sex, age</td><td>Male or female, age 18–65 years</td><td>Male, age 18–50 years</td><td>Male or female, age 18–65 years</td><td>Male or female, age 18–65 years</td></tr>
<tr><td>BMI and body weight</td><td>18–30 kg/m²</td><td>18–30 kg/m² and total body weight 65–88 kg</td><td>18–30 kg/m²</td><td>18–40 kg/m²</td></tr>
<tr><td>Confirmation of AD diagnosis, or specific AD symptom, or health status at baseline</td><td>Diagnosis of chronic AD for ≥1 year since first diagnosis, per Hanifin and Rajka Criteria:[4]
A. EASI ≥12 at screening and ≥16 at baseline (Day 1, predose).
B. IGA score ≥3 (IGA 0–4 scale:</td><td>Judged to be in good health based on medical history, physical examination, vital signs, 12-lead electrocardiogram, and laboratory profile at screening.</td><td>Diagnosis of AD for ≥1 year since first diagnosis, per Hanifin and Rajka Criteria[4] (see study 1 for full criteria).</td><td>Diagnosis of AD for ≥1 year since first diagnosis, per the Hanifin and Rajka Criteria[4] (see study 1 for full criteria).

Plus, ≥1 AD lesion for which treatment with</td></tr>
</table>

	3 = moderate;<br>4 = severe) at screening and at baseline. Greater than or equal to 10% body surface area of AD involvement at screening.<br>C. Willingness to continue stable use of an additive free, basic, bland emollient twice daily for ≥7 days before baseline and throughout the study.<br>D. Candidate for systemic therapy and history of inadequate response or contraindication to topical corticosteroids and/or topical calcineurin inhibitors prior to screening, per the investigator’s opinion.			medium potency TCS is indicated.

**Medication use in healthy male study participants**	N/A	Discontinuation of all medications (including over-the-counter medications and herbal supplements) except occasional paracetamol (maximum dose of 2 g/day and maximum of 10 g/2 weeks) ≥2 weeks prior to first administration of study medication.	N/A	N/A
**Recreational drug use in healthy male study participants**	N/A	Negative urine drug screen (amphetamines, barbiturates, benzodiazepines, cannabis, cocaine, opiates, methadone, and tricyclic antidepressants) and alcohol breath test.	N/A	N/A
**Smoking status and use of nicotine products in healthy male study participants**	N/A	Non-smoker (individual who abstained from smoking or nicotine-containing products for ≥1 year prior to first dosing), not currently using any nicotine-containing products.	N/A	N/A

AD, atopic dermatitis; BMI, body mass index; EASI, eczema area and severity index; IGA, Investigator Global Assessment; N/A, not available; TCS, topical corticosteroids

Exclusion criteria

Supplementary Table S2: Full exclusion criteria, studies 1–4

<table>
<tr><th>Exclusion criteria</th></tr>
<tr><td>

All studies, unless otherwise stated:

- Known hypersensitivity to MOR106 ingredients, as determined by the investigator (e.g. anaphylaxis requiring hospitalization).
- Prior treatment with MOR106.
- Positive serology for hepatitis B (positive hepatitis core antibody and/or positive hepatitis B surface antigen), or hepatitis C (anti-HCV), or any history of hepatitis from any cause with the exception of hepatitis A (***all studies***) and B (***Study 4 only***). Study participants immune to hepatitis B through vaccination were included.
- Study participants with a history of varicella zoster virus or ≥1 episode of herpes zoster or herpes zoster infection within one year of screening (study participants with history of herpes simplex types 1 and 2 and vaginal candidiasis were permitted).
- Any concurrent illness, condition, disability, or clinically significant abnormality (including laboratory tests, New York Heart Association classification ≥III) or clinically significant illness in the 3 months prior to initial study drug administration that, in the investigator's opinion represented a safety risk for participation, or may affect interpretation of clinical data, or may prevent the study participant from safely completing assessments required by the protocol.
- Any of the following laboratory findings:
    - Hemoglobin <12 g/dL (male) or <10 g/dL for female (***studies 2–4***).
    - White blood cell count <3.0 x 10^9 cells/L.
    - Neutrophil count <1.5 x 10^9 cells/L.
    - Platelet count <100 x 10^9 cells/L.
    - Serum alanine aminotransferase or aspartate aminotransferase >2 x ULN.
    - Total bilirubin level >1.5 x ULN (***studies 2–4***).
        - In study participants with Gilbert's syndrome, values of other liver enzymes (e.g. alkaline phosphatase, GGT and ALT/AST should be within normal range, the total bilirubin should not exceed 85 µmol/L [equals 5 mg/dL] and the level of conjugated bilirubin should not be >20% of total bilirubin, ***studies 2 and 3***).
    - Creatinine clearance <80 mL/min according to the Cockcroft formula (***studies 2 and 3***).

</td></tr>
</table>

• History of malignancy within 5 years prior to screening, with the exception of excised and curatively treated non-metastatic basal cell carcinoma or squamous cell carcinoma of the skin (***all studies***), carcinoma *in situ* of the cervix or of the breast (***Study 1***) and prostate cancer T1a or T1b using the TNM (tumor, nodes, metastasis) clinical staging system (***study 1***).<br>• Clinically significant abnormalities at the discretion of the investigator detected on vital signs or physical examination (other than AD) at screening or baseline (Day 1, predose).<br>• Receipt of an attenuated vaccination within 4 weeks of baseline, or expected to receive one during the study.<br>• Investigator or any sub-investigator, research assistant, pharmacist, study coordinator, or other staff or relative thereof who is directly involved in the conduct of the study.<br>• Use of any prohibit medicines in **Supplementary Table S3**				
	**Study 1**	**Study 2**	**Study 3**	**Study 4**
**Location of AD lesions**	N/A	N/A	N/A	AD lesions located predominantly (≥50% of the cumulative lesion area) on face and genital areas
**Immune system**	Known or suspected history of immunosuppression (e.g. HIV infection), including history of invasive opportunistic infections (e.g. tuberculosis, histoplasmosis, listeriosis, coccidioidomycosis, pneumocystosis, aspergillosis) despite infection resolution or unusually frequent, recurrent, or prolonged	History of/current immunosuppressive condition (e.g. HIV infection).<br><br>Current active, latent or history of tuberculosis infection, based on medical history and as determined by a positive QuantiFERON TB Gold test at screening (for indeterminate results, one retest was permitted and if the	Known or suspected history of immunosuppression (e.g. HIV infection), including history of invasive opportunistic infections (e.g. tuberculosis, histoplasmosis, listeriosis, coccidioidomycosis, pneumocystosis, aspergillosis) despite infection resolution or unusually frequent, recurrent, or prolonged	Known or suspected history of immunosuppression (e.g. HIV infection), including history of invasive opportunistic infections (e.g. tuberculosis, histoplasmosis, listeriosis, coccidioidomycosis, pneumocystosis, aspergillosis) despite infection resolution or unusually frequent, recurrent, or prolonged

	infections, per investigator judgment.	study participant remained indeterminate following a retest, the study participant was excluded).	infections, per investigator judgment.	infections, per investigator judgment.
**Pregnancy and breastfeeding**	Pregnant or breast feeding female, or female planning to become pregnant.	Pregnant or breastfeeding partner, or a partner intending to become pregnant or to breastfeed.	Male with a pregnant or breastfeeding partner, or a partner intending to become pregnant or to breastfeed.	Pregnant or breast feeding female, or female planning to become pregnant.
**Prohibited drugs and treatments**	Receipt of any of the following treatments:<br>A. Exposure to a biologic therapy for AD<br>B. Immunosuppressive / immunomodulating drugs (e.g. systemic corticosteroids, cyclosporine, mycophenolate-mofetil, interferon-gamma, azathioprine, methotrexate) within 4 weeks of baseline<br>C. Phototherapy (ultraviolet B, or psoralen and ultraviolet A) for AD within 4 weeks of baseline	Treatment with any drug known to have a well-defined potential for toxicity to a major organ in the 3 months prior to the first dose of study drug.	Treatment with any drug known to have a well-defined potential for toxicity to a major organ in the 3 months prior to the first dose of study drug<br><br>***Use of prohibited treatments A–F listed for study 1***	Prior exposure to Dupilumab<br><br>***Use of prohibited treatments B–F listed for study 1***

	D. Treatment with topical corticosteroids or topical calcineurin inhibitors within 2 weeks of baseline<br>E. Treatment with biologics (for non-atopic dermatitis indications within 5 half-lives [if known] or 12 weeks prior to baseline [if unknown])<br>F. Regular use (>2 visits/week) of a tanning booth/parlor within 4 weeks of screening			
**Infections**	Active chronic or acute infection requiring treatment with systemic (oral, s.c. or i.v.) antibiotics, antivirals, antiparasitics, antiprotozoals, or antifungals, within 4 weeks of baseline, or clinical signs of infective eczema within 1 week before baseline. Note: study participants may	N/A	Active chronic or acute infection requiring treatment with systemic (oral, s.c. or i.v.) antibiotics, antivirals, antiparasitics, antiprotozoals, or antifungals, within 4 weeks of baseline, or clinical signs of infective eczema within 1 week before baseline. Note: study participants may	Active chronic or acute infection requiring treatment with systemic (oral, s.c. or i.v.) antibiotics, antivirals, antiparasitics, antiprotozoals, or antifungals, within 4 weeks of baseline, or clinical signs of infective eczema within 1 week before baseline. Note: study participants may

	be rescreened after infection resolves.		be rescreened after infection resolves.<br><br>Oral fluconazole for vaginal yeast infection and oral antiviral medication for herpes labialis and herpes genitalis was permitted.	be rescreened after infection resolves.<br><br>Oral fluconazole for vaginal yeast infection and oral antiviral medication for herpes labialis and herpes genitalis was permitted.
**Experimental therapies**	N/A	Participation in another experimental therapy study within 90 days or five times the half-life of the experimental therapy, whichever is longer, prior to dosing for this study, or current enrolment in any other study.	Participation in another experimental therapy study within 90 days or five times the half-life of the experimental therapy, whichever is longer, prior to dosing for this study, or current enrolment in any other study.	Participation in another experimental therapy study within 90 days or five times the half-life of the experimental therapy, whichever is longer, prior to dosing for this study, or current enrolment in any other study.
**Electronic diary competence**	Not able to manage the electronic diary (e-diary) as per assessment of the investigator.	N/A	Not able to manage the electronic diary (e-diary) as per assessment of the investigator.	Not able to manage the electronic diary (e-diary) as per assessment of the investigator.
**Recreational drug/alcohol use**	N/A	Active drug or alcohol abuse (more than an average of three glasses of wine or beer or equivalent/day) within 2 years prior to the initial IMP administration.	Active drug or alcohol abuse (more than an average of three glasses of wine or beer or equivalent/day) within 2 years prior to the initial IMP administration.	Active drug or alcohol abuse.

**Blood loss**	N/A	Significant blood loss (including blood donation >450 mL), or had a transfusion of any blood product within 12 weeks prior to the first dose of study drug.	Significant blood loss (including blood donation >450 mL), or had a transfusion of any blood product within 12 weeks prior to the first dose of study drug.	N/A
**Eczema**	History of eczema herpeticum in the last 12 months prior to screening	N/A	N/A	N/A
**Miscellaneous**	N/A	N/A	N/A	Any condition or circumstances that in the opinion of the investigator may make a study participant unlikely or unable to complete the study or comply with study procedures and requirements.

AD, atopic dermatitis; ALT, alanine aminotransferase; AST, aspartate aminotransferase; GGT, glutamyl transferase; HCV, hepatitis C virus; HIV, human immunodeficiency virus; IMP, investigational medicinal product; i.v., intravenous; s.c., subcutaneous; TB, tuberculosis; TNM, tumor node metastasis; ULN, upper limit of normal

Supplementary Table S3: List of prohibited medicines and therapies

**Studies in patients with AD (studies 1,3 and 4)**	Exposure to a biologic therapy for AD
	Immunosuppressive/ immunomodulating drugs (e.g. systemic corticosteroids, cyclosporine, mycophenolate-mofetil, IFN-γ, azathioprine, methotrexate, etc.) within 4 weeks of baseline
	Phototherapy (UVB) or PUVA) for AD within 4 weeks of baseline
	Treatment with TCS or TCI within 2 weeks (studies 1 and 3) or 1 week (study 4) prior to baseline
	Treatment with biologics (for non-AD indications) within five half-lives (if known) or 12 weeks prior to baseline visit, whichever is longer
	Regular use (>2 visits per week) of a tanning booth/parlor within 4 weeks of screening
**Study 2**	Consumption of a large quantity of coffee, tea (>6 cups/day) or equivalent
**Study 2 and 3**	Treatment with any drug known to have a well-defined potential for toxicity to a major organ in the 3-months prior to first dose of study drug
	Active drug or alcohol abuse (more than an average of three glasses of wine, or beer, or equivalent/day) within 2 years prior to first dose of study drug
**Study 4**	Prior exposure to dupilumab

AD, atopic dermatitis; IFN-γ, interferon-γ; PUVA, Psoralen Ultraviolet A; TCI, topical calcineurin inhibitors; TCS, topical corticosteroids; UVB, ultraviolet.

Secondary endpoints

Due to early termination, not all endpoints were assessed as planned – for more information refer to the Efficacy Analyses in the Statistical Operations section, below.

Study 1

Percentage of patients that achieved ≥50% improvement in eczema area and severity index (EASI) score from baseline (EASI50) at Day 85 and all timepoints, percentage of patients that achieved an Investigators' Global Assessment (IGA) score of 0 or 1 at Day 85 and at all timepoints, percentage of patients that achieved an IGA score of ≥2 at Day 85 and all timepoints, percent change in Scoring Atopic Dermatitis (SCORAD) score from baseline to Day 85 and at all timepoints, incidence of treatment-emergent adverse events (TEAEs), adverse events of special interest (AESIs), serious adverse events (SAEs), and discontinuations due to adverse events (AEs), and characterization of MOR106 immunogenetic and pharmacokinetic (PK) profiles from baseline to Day 197 or early discontinuation.

Study 2

Occurrence of anti-drug antibodies (ADA) from baseline to Day 50 or early discontinuation.

Study 3

Occurrence of ADA from baseline through Day 197 or early discontinuation (ED), MOR106 serum concentrations after multiple subcutaneous (s.c.) administrations, percent change in EASI score from baseline to Day 85, percent of patients that achieved EASI50 at Day 85, time to first response of EASI50, percentage of patients that achieved ≥75% and ≥90% improvement in EASI score from baseline to Day 85, percentage of patients that achieved an IGA score of 0 or 1 at Day 85, percentage of

patients who achieved an IGA score reduction of ≥2 at Day 85, percent change in SCORAD score from baseline to Day 85, absolute and percent change in body surface area, absolute and percent change in Patient Orientated Eczema Measure (POEM) score from baseline to Day 85 and weekly change from baseline in pruritus numeric rating score (NRS).

Study 4

MOR106 serum concentrations after repeated s.c. administrations and occurrence of ADA from baseline through Day 169 or early discontinuation.

Settings and locations

Study 1 was conducted at 40 centers (enrolling sites) in Hungary, Germany, United Kingdom (UK) and Poland. In addition, four sites screened patients without enrolment. Study 2 was conducted at one center in the UK. Study 3 was conducted at 15 centers in four countries (Germany, Spain, UK, and Ukraine). Study 4 was conducted at 23 centers in the United States (of which 16 sites screened patients and 14 randomized patients), and in one center in Canada (no patient recruited).

Assigning individuals to treatment groups

In all studies, the coordinating investigator in each country was responsible for the enrolment and randomization of study participants in that location (coordinating investigators are stated under “Acknowledgements”, main text). Eligible study participants were assigned a participant randomization number in ascending order by a study investigator. Allocation to a given treatment was performed using a

computerized interactive web response system, according to a prespecified randomization scheme prepared by an independent statistician.

Blinding and unblinding

Studies 1, 3 and 4 were double-blind clinical studies. The study participant, investigator, clinical site staff, sponsor, and entire clinical study processing team remained blinded to treatment assignment. Only site staff (pharmacist and/or other appropriate qualified staff) were unblinded to enable drug preparation according to the study participant's assigned randomization number. Blinding could be broken only if the investigator deemed it necessary for reasons of safety. Study 2 was open label.

Concomitant medications

In patients with atopic dermatitis (AD), unless specifically prohibited, concomitant medications including nasal/inhaled corticosteroids were permitted. Biologics were discontinued 12-weeks, systemic drugs 4-weeks, and topical therapies 1-week (study 4) or 2-weeks (studies 1 and 3) prior to baseline. For full details of prohibited medications and therapies, see **Supplementary Table S3**. In healthy male study participants, all medication (over the counter and prescription) was discontinued ≥2-weeks/≥5-half-lives (whichever longer) prior to MOR106 administration.

Efficacy assessments

EASI was assessed per Hanifin et al. (2001)[5], Investigator Global Assessment (IGA) per Rehal et al. (2011)[6] and a patient-reported pruritus NRS was completed every

morning and evening using an electronic device (11-point scale: 0, no itch; 10, worst itch imaginable).

Safety assessments

AEs were graded according to common terminology criteria for AEs. Investigators followed up on SAEs until the event subsided/disappeared, stabilized, was otherwise explained, or the study participant was lost to follow up. AESIs were defined as: skin-related AEs except exacerbation and infective exacerbation of AD (studies 1, 3 and 4), or infusion-related reactions (IRR) of grade ≥2, or injection site reactions (ISRs) of grade ≥3.

PK, pharmacodynamic (PD) and immunogenicity assessments

PK serum samples were analyzed using a validated electrochemiluminescence-based ligand binding assay, with a lower limit of detection of 50 ng/mL (MorphoSys AG, Germany). PK data in study 2 were evaluated by non-compartmental data analysis and, in combination with data from the first-in-human study[7], were used to establish a PK model. Serum concentrations obtained in studies 1, 3 and 4 were compared with model-simulated profiles.

The PD parameter 'target engagement' was assessed via total serum interleukin (IL)-17C determination (free IL-17C and IL-17C bound to MOR106) throughout studies 1, 3 and 4. The assessment was performed using a validated electrochemiluminescence-based ligand binding assay with a lower limit of detection of 80 pg/mL (MorphoSys AG, Germany).

Immunogenicity was determined in all studies via absolute and semi-quantitative determination of ADA and serum samples were analyzed for ADA using a validated electrochemiluminescence-based ligand binding assay (MorphoSys AG, Germany).

Statistical operations

Statistical analyses were performed using SAS® version 9.4 (Cary, NC, USA). Inferential statistics were interpreted at the 2-sided, 5% significance level. Study participant disposition, demographics, baseline characteristics, medical history, use of study drug and prior and concomitant therapies were analyzed descriptively. For each study, three analysis sets were collated: a safety analysis set, for safety analyses including immunogenicity (randomized study participants who received ≥1 dose of study drug); a full analysis set, for efficacy analyses (randomized study participants who received ≥1 dose and had at ≥1 post-baseline efficacy assessment); and a PK analysis set, for analysis of PK (subset of the safety analysis set with evaluable MOR106 serum concentration).

Efficacy analyses

Study 1

Due to the early termination of the MOR106 clinical program in AD:

- The primary analysis of study 1 was not performed once all patients had completed Day 85 (end of treatment) or discontinued, but at Day 197 (end of follow up); patients without a Day 85 value did not contribute to the efficacy analysis set.

- No sensitivity analyses with formal missing data imputation were performed for EASI score.
- Time-to-event data was only listed.
- EASI75, EASI90 and terminal half-life ($T_{1/2}$) were not derived.
- Antibody-complexed IL-17C concentration was based on the safety analysis set.

The primary endpoint was analyzed using a mixed effects model for repeated measures that included treatment and visit as fixed effects, baseline EASI score and country as covariates and treatment-visit as an interaction term. For the achievement of EASI50, binary parameters were analyzed using frequency tabulations and a generalized estimating equations model that included treatment and visit as fixed effects, baseline score and country as covariates and treatment-visit as an interaction term.

Study 3

Due to the early termination of the MOR106 clinical program in AD:

- The primary analysis for all patients that completed their Day 85 visit or discontinued earlier was not conducted as planned; instead, the analysis of the primary endpoint was conducted after all patients had completed their study participation.
- The sensitivity analyses with formal missing data imputation for EASI score and the time-to-event analysis were not performed.

- EASI75, EASI90, the percent of patients with an IGA score reduction of ≥2, changes in POEM score from baseline and terminal half-life ($T_{1/2}$) were not derived.

Per sponsor's decision, independent of study termination:

- Safety analyses were based on the randomized treatment and not on the actual treatment.

Study 4

Due to the early termination of the MOR106 clinical program in AD:

- The Primary Analysis when all patients completed their Day 57 visit was not conducted.
- The sensitivity analyses with formal missing data imputation for EASI score, and the time-to-event analysis were not performed.
- EASI75 and EASI90, the percentage of patients with an IGA score reduction of ≥2, and terminal half-life ($T_{1/2}$) were not derived.

Per sponsor's decision, independent of termination of treatment phase:

- The full analysis set (FAS) population definition was updated to be the same as the safety analysis set (due to the type of patients and disease, it was not anticipated to have a difference between these populations).
- The safety analyses were based on the randomized treatment and not on actual treatment.

Rescue medication

In all studies, if rescue medication (i.e. treatment not per protocol, including topical corticosteroid [TCS] initiation [study 1 and 3]/escalation [study 4]) was required during treatment, study participants were discontinued from randomized treatment. Data collected after administration of rescue medication were excluded from efficacy analyses. In studies 1 and 3, TCS and topical calcineurin inhibitors (TCI) were permitted for intolerable symptoms of AD during the off-treatment follow up at the investigators' discretion.

Handling of missing data

In study 1, multiple imputation was used to handle missing data; in studies 2–4, no imputations were performed. Pairwise comparisons were explored without multiplicity correction.

Sample size determination

Study 1

Sample size was determined to adequately support efficacy analyses of the primary endpoint and a robust population-based PK versus clinical efficacy model. For the primary endpoint, a maximum effect size of 40%, a standard deviation of 50% on EASI change from baseline, a 2-sided significance level of 5% and a dropout rate of 15% determined 30 patients per arm were required to provide >85% power. PK–efficacy model simulations predicted substantially decreased interindividual variability for estimates of half maximal effective concentration (EC50) with 45 patients in the MOR106 Q2W and placebo treatment arms and the sample size of these arms was increased accordingly.

Study 2

Strict statistical criteria were not used to determine sample size. Enrolment of 32 healthy male study participants was planned for reasonable precision around the estimates derived for safety, PK and PD evaluation.

Study 3

Strict statistical criteria were not used to determine sample size. Enrolment of 45 patients with AD was planned. An assumed dropout rate of approximately 15% was assumed for n=39 evaluable patients for efficacy analysis and reasonable precision for estimates derived for safety, PK, efficacy and PD evaluation. For the key efficacy endpoint (change from baseline in EASI score at Day 85) with 39 evaluable patients (MOR106, n=26; placebo, n=13) and a common standard deviation of 50%, there would be 63% power to detect a 40% treatment effect. The half-width of the 95% confidence interval (CI) around the difference from placebo would be ~33%. For the safety objective of the study, if no treatment-emergent SAE was observed in the sample of 26 patients treated with MOR106, it could be concluded with 95% confidence that the true incidence of treatment-emergent SAEs would be <11%.

Study 4

Strict statistical criteria were not used to determine sample size. Enrolment of 60 patients (MOR106, n=40; placebo, n=20) was planned, for an estimated 51 evaluable patients and reasonable precision around estimates derived for safety, PK, PD, and efficacy evaluation. With a sample size of 40 patients on MOR106, there

was 87% probability to observe ≥1 occurrence of an AE (assuming a natural incidence of 5%). The control group served as internal validation for any potential finding. For the efficacy endpoint (change from baseline in EASI score at Day 57), with a total of 51 evaluable patients and 15% dropout at Day 57, and an assumed common standard deviation of 50%, there was 51% power to detect a 30% treatment effect versus placebo (using a two-sample t-test). The half-width of the 95% confidence interval around the difference from placebo would be ~30%.

Statistical model for relative bioavailability and dose-proportionality

In study 2, relative bioavailability was assessed using a mixed effect model with treatment (4 mg/kg s.c. and 4 mg/kg intravenous [i.v.] treatment groups only) as a fixed effect and study participant as random effect on log-transformed maximum serum concentration (C_{max}), area under the curve $(AUC)_{0-t}$ and $AUC_{0-\infty}$ values. Point estimates were calculated as the geometric mean ratios for each parameter, for the 4 mg/kg s.c. relative to the 4 mg/kg i.v. treatment groups. Dose-proportionality across the s.c. dose range was tested using a power model approach. A mixed-effects model with log-transformed dose as a continuous fixed effect and study participant as random effect was applied to log-transformed C_{max} and AUC_{0-t} and $AUC_{0-\infty}$ values.

Additional results

Study participant disposition and dates of study conduct

Supplementary Figure S1. Study participant disposition and dates of study conduct, studies 1–4

Study 1

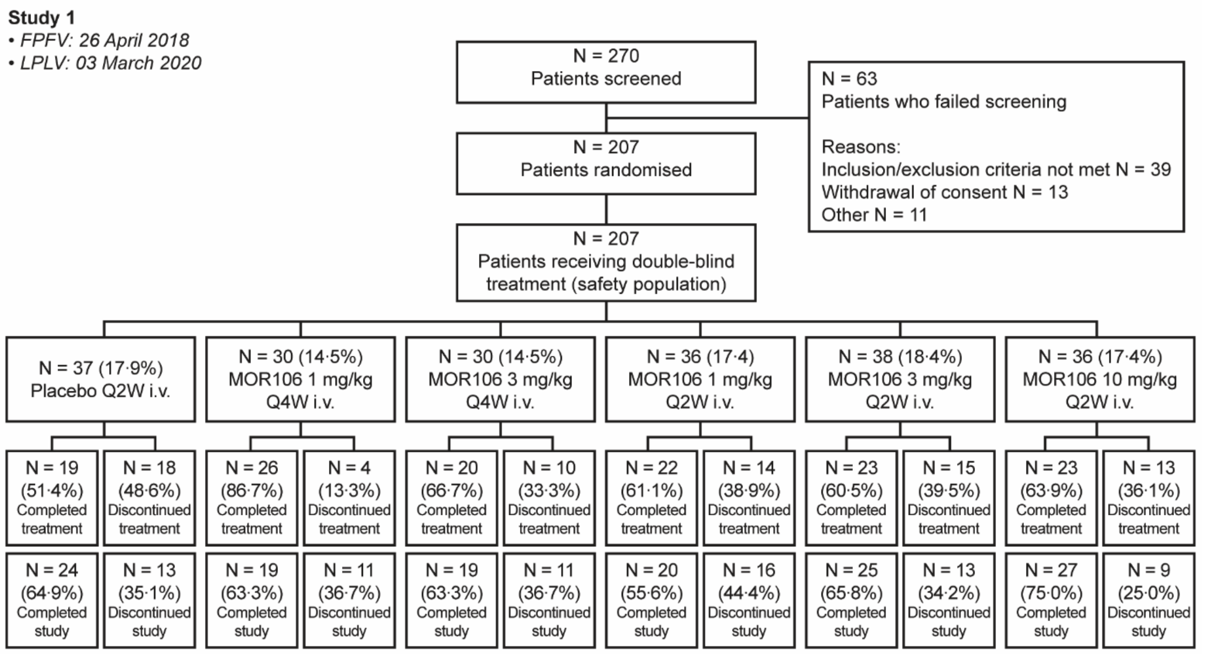

Reasons for discontinuation in Study 1:

	Placebo Q2W i.v. (n=37)	MOR106 1 mg/kg Q4W i.v. (n=30)	MOR106 3 mg/kg Q4W i.v. (n=30)	MOR106 1 mg/kg Q2W i.v. (n=36)	MOR106 3 mg/kg Q2W i.v. (n=38)	MOR106 10 mg/kg Q2W i.v. (n=36)
**Discontinued treatment**	18 (48·6)	4 (13·3)	10 (33·3)	14 (38·9)	15 (39·5)	13 (36·1)
**Adverse event**	3 (8·1)	3 (10)	4 (13·3)	4 (11·1)	1 (2·6)	1 (2·8)
**Protocol deviation**	1 (2·7)	0	0	0	0	1 (2·8)
**Physician's decision**	1 (2·7)	0	1 (3·3)	1 (2·8)	0	0
**Lack of efficacy**	2 (5·4)	1 (3·3)	2 (6·7)	1 (2·8)	0	2 (5·6)
**Withdrawal by patient**	6 (16·2)	0	2 (6·7)	3 (8·3)	7 (18·4)	5 (13·9)
**Protocol-specified withdrawal criterion**	1 (2·7)	0	0	0	0	0
**Other**	4 (10·8)	0	1 (3·3)	5 (13·9)	7 (18·4)	4 (11·1)
**Discontinued study**	13 (35·1)	11 (36·7)	11 (36·7)	16 (44·4)	13 (34·2)	9 (25·0)
**Adverse event**	1 (2·7)	3 (10)	5 (16·7)	4 (11·1)	2 (5·3)	1 (2·8)
**Lost to follow up**	0	1 (3·3)	0	1 (2·8)	0	0
**Physician's decision**	1 (2·7)	0	1 (3·3)	0	1 (2·6)	0
**Lack of efficacy**	0	0	1 (3·3)	0	0	1 (2·8)
**Withdrawal by patient**	9 (24·3)	5 (16·7)	4 (13·3)	9 (25·0)	9 (23·7)	6 (16·7)
**Other**	2 (5·4)	2 (6·7)	0	2 (5·6)	1 (2·6)	1 (2·8)

Study 2

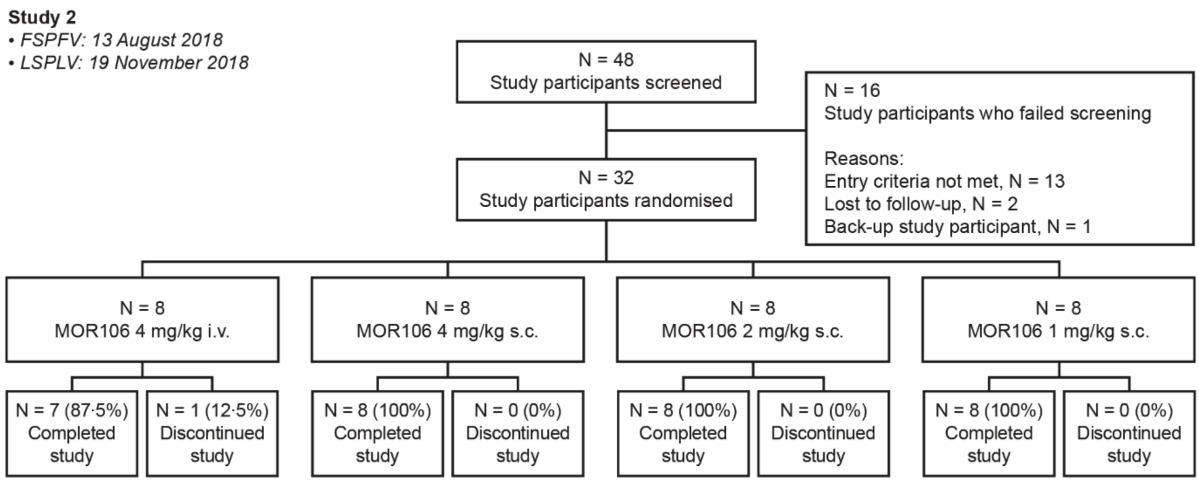

Reasons for discontinuation in Study 2:

One study participant in the MOR106 4 mg/kg i.v. group requested to be discontinued on Day 4 and agreed to have an early discontinuation visit on Day 13. No other study participants discontinued.

Study 3

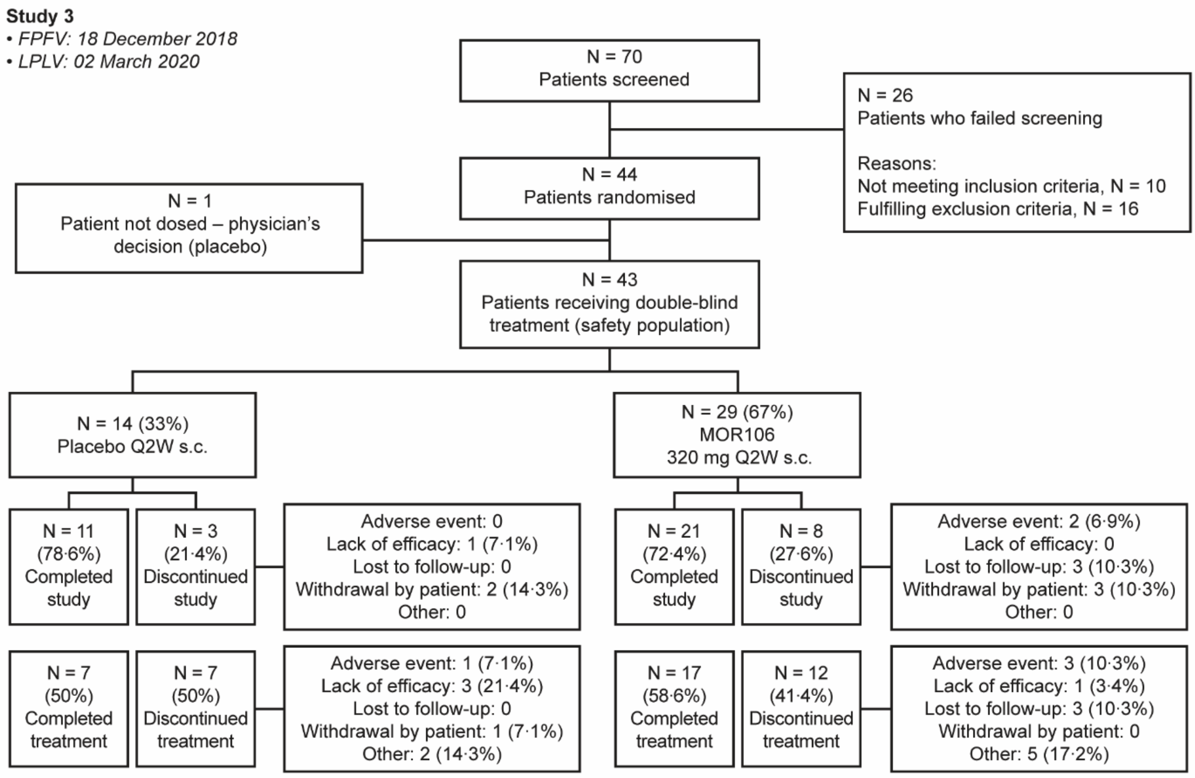

Study 4

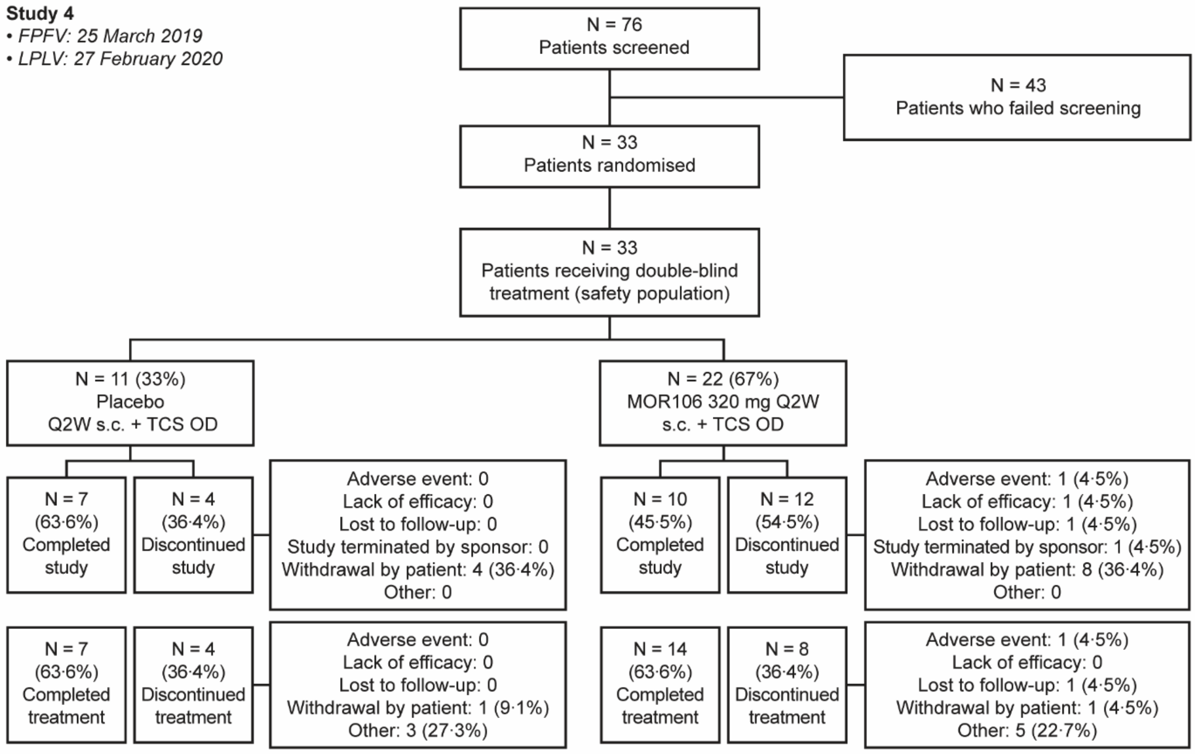

FPFV, first patient, first visit; FSPFV, first study participant, first visit; i.v., intravenous; LPLV, last patient, last visit; LSPLV, last study participant, last visit; OD, once-daily; Q2W, every 2 weeks; Q4W, every 4 weeks; s.c., subcutaneous; TCS, topical corticosteroids

Adherence to planned dosing schedules

Patients with moderate–severe AD

Supplementary Table S4: Number of infusions/injections received, patients with moderate–severe AD

Study 1							
**Number of infusions received, n (%)**	**Placebo Q2W i.v. (n=37)**	**MOR106 1 mg/kg Q4W i.v. (n=30)**	**MOR106 3 mg/kg Q4W i.v. (n=30)**	**MOR106 1 mg/kg Q2W i.v. (n=36)**	**MOR106 3 mg/kg Q2W i.v. (n=38)**	**MOR106 10 mg/kg Q2W i.v. (n=36)**	**Total (n=207)**
**6**	19 (51·4)	26 (86·7)	20 (66·7)	22 (61·1)	23 (60·5)	23 (63·9)	133 (64·3)
**5**	4 (10·8)	0	0	4 (11·1)	2 (5·3)	1 (2·8)	11 (5·3)
**4**	2 (5·4)	0	3 (10·0)	2 (5·6)	5 (13·2)	2 (5·6)	14 (6·8)
**3**	3 (8·1)	1 (3·3)	4 (13·3)	3 (8·3)	2 (5·3)	5 (13·9)	18 (8·7)
**2**	5 (13·5)	3 (10·0)	1 (3·3)	4 (11·1)	5 (13·2)	4 (11·1)	22 (10·6)
**1**	4 (10·8)	0	2 (6·7)	1 (2·8)	1 (2·6)	1 (2·8)	9 (4·3)

Study 3			
**Number of days with injections received, n (%)**	**Placebo Q2W s.c. (n=14)**	**MOR106 320 mg Q2W s.c. (n=29)**	**Total (n=43)**
**6**	7 (50·0)	17 (58·6)	24 (55·8)
**5**	0	1 (3·4)	1 (2·3)
**4**	1 (7·1)	2 (6·9)	3 (7·0)
**3**	0	4 (13·8)	4 (9·3)
**2**	4 (28·6)	2 (6·9)	6 (14·0)
**1**	2 (14·3)	3 (10·3)	5 (11·6)
**Study 4**			
**Number of days with injections received, n (%)**	**Placebo Q2W s.c. + TCS OD (n=11)**	**MOR106 320 mg Q2W s.c. +TCS OD (n=22)**	**Total (n=33)**
**4**	7 (63·3)	14 (63·6)	21 (63·6)
**3**	1 (9·1)	0	1 (3·0)
**2**	2 (18·2)	5 (22·7)	7 (21·2)
**1**	1 (9·1)	3 (13·6)	4 (12·1)

Data are per the full analysis set.
AD, atopic dermatitis; i.v. intravenous; OD, once-daily; Q2W, every two weeks; Q4W, every four weeks; s.c., subcutaneous; TCS, topical corticosteroids

Healthy male study participants (Study 2)

In study 2, all randomized study participants received one dose as planned (N=8 in each treatment group: MOR106 1 mg/kg s.c.; MOR106 2 mg/kg s.c; MOR106 4 mg/kg s.c.; MOR106 4 mg/kg i.v.).

Additional safety data, patients with moderate–severe AD

Supplementary Table S5: TEAEs by SOC and PT with overall incidence ≥5% in patients with moderate–severe AD, study 1

	**Study 1 (MOR106/placebo administered by i.v. infusion)**					
**Data are n (%)**	**Placebo Q2W (n=37)**	**MOR106 1 mg/kg Q4W (n=30)**	**MOR106 3 mg/kg Q4W (n=30)**	**MOR106 1 mg/kg Q2W (n=36)**	**MOR106 3 mg/kg Q2W (n=38)**	**MOR106 10 mg/kg Q2W (n=36)**
**Any TEAE**	26 (70·3)	27 (90·0)	22 (73·3)	22 (61·1)	29 (76·3)	26 (72·2)
**Skin and subcutaneous tissue disorders**						
**Dermatitis atopic**	11 (29·7)	11 (36·7)	14 (46·7)	10 (27·8)	13 (34·2)	16 (44·4)
**Acne**	0	1 (3·3)	2 (6·7)	0	1 (2·6)	2 (5·6)
**Pruritis**	0	1 (3·3)	2 (6·7)	0	0	2 (5·6)
**Dermatitis**	0	1 (3·3)	0	1 (2·8)	2 (5·3)	0
**Infections and infestations**						
**Nasopharyngitis**	7 (18·9)	4 (13·3)	1 (3·3)	5 (13·9)	5 (13·2)	5 (13·9)
**Upper respiratory tract infection**	2 (5·4)	3 (10·0)	1 (3·3)	3 (8·3)	1 (2·6)	2 (5·6)
**Folliculitis**	0	0	1 (3·3)	2 (5·6)	3 (7·9)	3 (8·3)
**Pharyngitis**	1 (2·7)	0	1 (3·3)	1 (2·8)	0	3 (8·3)
**Sinusitis**	3 (8·1)	1 (3·3)	1 (3·3)	0	0	0
**Bronchitis**	2 (5·4)	0	0	1 (2·8)	0	1 (2·8)
**Conjunctivitis**	0	0	0	0	1 (2·6)	3 (8·3)
**Superinfection**	0	2 (6·7)	0	0	0	0
**Respiratory, thoracic and mediastinal disorders**						
**Asthma**	3 (8·1)	0	0	0	2 (5·3)	2 (5·6)
**Rhinitis allergic**	1 (2·7)	1 (3·3)	2 (6·7)	0	0	1 (2·8)
**Rhinorrhea**	0	1 (3·3)	0	1 (2·8)	2 (5·3)	1 (2·8)
**Oropharyngeal pain**	0	1 (3·3)	0	1 (2·8)	2 (5·3)	0
**Nervous system disorders**						
**Headache**	4 (10·8)	3 (10·0)	6 (20·0)	1 (2·8)	3 (7·9)	1 (2·8)
**Investigations**						
**Aspartate aminotransferase increased**	1 (2·7)	1 (3·3)	0	0	2 (5·3)	1 (2·8)
**Alanine aminotransferase increased**	2 (5·4)	1 (3·3)	0	0	1 (2·6)	0
**C-reactive protein increased**	1 (2·7)	0	0	0	0	2 (5·6)
**Blood creatinine increased**	1 (2·7)	0	0	2 (5·6)	0	0
**Gammaglutamyltransferase increased**	0	2 (6·7)	0	0	1 (2·6)	0

**Musculoskeletal and connective tissue disorders**						
**Arthralgia**	2 (5·4)	0	0	1 (2·8)	0	1 (2·8)
**Myalgia**	1 (2·7)	1 (3·3)	0	0	0	2 (5·6)
**Back pain**	0	2 (6·7)	0	0	0	2 (5·6)
**General disorders and administration site conditions**						
**Oedema peripheral**	1 (2·7)	1 (3·3)	0	2 (5·6)	1 (2·6)	2 (5·6)
**Influenza like illness**	0	0	0	0	2 (5·3)	0
**Metabolism and nutrition disorders**						
**Hypertriglyceridemia**	1 (2·7)	0)	0	0	1 (2·6)	3 (8·3)
**Blood and lymphatic system disorders**						
**Lymphadenopathy**	0	0	0	0	2 (5·3)	0
**Psychiatric disorders**						
**Insomnia**	2 (5·4)	0	0	0	0	0
**Vascular disorders**						
**Thrombophlebitis**	2 (5·4)	0	0	0	0	0

i.v. intravenous; PT, preferred term; Q2W, every 2 weeks; Q4W, every 4 weeks; s.c., subcutaneous; SOC, system organ class; TEAE, treatment-emergent adverse event

Supplementary Table S6: TEAEs by SOC and PT with overall incidence ≥5% in patients with moderate–severe AD, study 3

Data are n (%)	Study 3<br>(Placebo and MOR106 administered by s.c. injection)<br>Placebo Q2W<br>(n=14)	 <br>MOR106 320 mg Q2W<br>(N=29)
**Any TEAE**	11 (78·6)	25 (86·2)
**Infections and infestations**		
**Nasopharyngitis**	1 (7·1)	5 (17·2)
**Influenza**	2 (14·3)	4 (13·8)
**Upper respiratory tract infection**	1 (7·1)	3 (10·3)
**Folliculitis**	0	2 (6·9)
**Rash pustular**	0	2 (6·9)
**Rhinitis**	2 (14·3)	2 (6·9)
**Laryngitis**	1 (7·1)	0
**Pharyngitis bacterial**	1 (7·1)	0
**Tinea pedis**	1 (7·1)	0
**Skin and subcutaneous tissue disorders**		
**Dermatitis atopic**	2 (14·3)	9 (31·0)
**Rash papular**	1 (7·1)	1 (3·4)
**Hyperhidrosis**	1 (7·1)	0
**Seborrheic dermatitis**	1 (7·1)	0
**Investigations**		
**Blood creatine phosphokinase increased**	0	2 (6·9)
**Alanine aminotransferase increased**	1 (7·1)	1 (3·4)
**Aspartate aminotransferase increased**	1 (7·1)	1 (3·4)
**Hepatic enzyme increased**	1 (7·1)	1 (3·4)
**Respiratory, thoracic and mediastinal disorders**		
**Oropharyngeal pain 1**	1 (7·1)	0
**Rhinorrhea 1**	1 (7·1)	0
**Upper-airway cough syndrome**	1 (7·1)	0
**General disorders and administration site conditions**		
**Feeling cold**	1 (7·1)	0
**Metabolism and nutrition disorders**		
**Hypertriglyceridemia**	1 (7·1)	1 (3·4)
**Immune system disorders**		
**Allergic oedema**	1 (7·1)	0
**Musculoskeletal and connective tissue disorders**		
**Musculoskeletal chest pain**	1 (7·1)	0
**Nervous system disorders**		
**Headache**	1 (7·1)	1 (3·4)
**Gastrointestinal disorders**		
**Gastroesophageal reflux disease**	2 (14·3)	0
**Hepatobiliary disorders**		
**Cholecystitis chronic**	1 (7·1)	0
**Injury, poisoning and procedural complications**		
**Post-traumatic pain**	1 (7·1)	0

**Sports injury**	1 (7·1)	0
**Renal and urinary disorders**		
**Proteinuria**	1 (7·1)	0

AD, atopic dermatitis; PT, preferred term; Q2W, every 2 weeks; s.c., subcutaneous; SOC, system organ class; TEAE, treatment-emergent adverse event

Supplementary Table S7: TEAEs by SOC and PT with overall incidence ≥5% in patients with moderate–severe AD, study 4

	**Study 4 (Placebo and MOR106 administered by s.c. injection)**	
**Data are n (%)**	**Placebo Q2W (n=11)**	**MOR106 320 mg Q2W (N=22)**
**Any TEAE**	5 (45·5)	11 (50·0)
**General disorders and administration site conditions**		
**Injection site reaction**	1 (9·1)	2 (9·1)
**Infections and infestations**		
**Folliculitis**	1 (9·1)	1 (4·5)
**Nasopharyngitis**	1 (9·1)	0
**Upper respiratory tract infection**	1 (9·1)	0
**Metabolism and nutrition disorders**		
**Hyperuricemia**	1 (9·1)	0
**Neoplasms benign, malignant and unspecified**		
**Squamous cell carcinoma**	1 (9·1)	0
**Skin and subcutaneous tissue disorders**		
**Skin exfoliation**	1 (9·1)	0

AD, atopic dermatitis; PT, preferred term; Q2W, every 2 weeks; s.c., subcutaneous; SOC, system organ class; TEAE, treatment-emergent adverse event

Supplementary Table S8: Skin related TEAEs by SOC and PT in patients with moderate–severe AD, studies 1, 3 and 4

	**Study 1 (MOR106/placebo administered by i.v. infusion)**						**Study 3 (MOR106/placebo administered by s.c. injection)**		**Study 4 (MOR106/placebo administered by s.c.)**	
**Data are n (%)**	**Placebo Q2W (n=37)**	**MOR106 1 mg/kg Q4W (n=30)**	**MOR106 3 mg/kg Q4W (n=30)**	**MOR106 1 mg/kg Q2W (n=36)**	**MOR106 3 mg/kg Q2W (n=38)**	**MOR106 10 mg/kg Q2W (n=36)**	**Placebo Q2W (n=14)**	**MOR106 320 mg Q2W (n=29)**	**Placebo Q2W + TCS OD (n=11)**	**MOR106 320 mg Q2W + TCS OD (n=22)**
**Any skin-related TEAE**	**1 (2·7)**	**4 (13·3)**	**3 (10·0)**	**1 (2·8)**	**7 (18·4)**	**8 (22·2)**	**3 (21·4)**	**9 (31·0)**	**2 (18·2)**	**1 (4·5)**
**Infections and infestations**	**1 (2·7)**	**1 (3·3)**	**2 (6·7)**	**1 (2·8)**	**3 (7·9)**	**5 (13·9)**	**1 (7·1)**	**7 (24·1)**	**1 (9·1)**	**1 (4·5)**
**Folliculitis**	0	0	1 (3·3)	1 (2·8)	3 (7·9)	3 (8·3)	0	2 (6·9)	1 (9·1)	1 (4·5)
**Rash pustular**	0	0	0	0	0	0	0	2 (6·9)	0	0
**Eczema herpeticum**	0	0	1 (3·3)	0	0	0	0	1 (3·4)	0	0
**Erysipelas**	0	0	0	0	0	1 (2·8)	0	0	0	0
**Eyelid infection**	0	0	0	0	1 (2·6)	0	0	0	0	0
**Herpes simplex**	0	0	0	0	0	0	0	1 (3·4)	0	0
**Furuncle**	0	1 (3·3)	0	0	0	0	0	0	0	0
**Soft tissue infection**	0	0	0	0	0	1 (2·8)	0	0	0	0
**Subcutaneous abscess**	0	0	0	0	0	0	0	1 (3·4)	0	0
**Superinfection bacterial**	1 (2·7)	0	0	0	0	0	0	0	0	0
**Tineas pedis**	0	0	0	0	0	0	1 (7·1)	0	0	0
**Skin and subcutaneous tissue disorders**	**0**	**2 (6·7)**	**2 (6·7)**	**0**	**3 (7·9)**	**2 (5·6)**	**2 (14·3)**	**2 (6·9)**	**1 (9·1)**	**0**
**Acne**	0	0 (3·3)	2 (6·7)	0	1 (2·6)	2 (5·6)	0	0	0	0
**Dermatitis contact**	0	1 (3·3)	0	0	1 (2·6)	0	0	0	0	0
**Dermatitis**	0		0	0	1 (2·6)	0	0	0	0	0
**Folliculitis**	0	1 (3·3)	0	0	0	0	0	0	0	0
**Rash**	0		0	0	0	0	0	1 (3·4)	0	0
**Rash erythematous**	0	1 (3·3)	0	0	0	0	0	0	0	0
**Rash papular**	0		0	0	0	0	1 (7·1)	10 (3·4)	0	0
**Seborrheic dermatitis**	0	1 (3·3)	0	0	0	0	1 (7·1)	0	0	0
**Skin exfoliation**	0		0	0	0	0	0	0	1 (9·1)	0
**Blood and lymphatic system disorders**	**0**	**0**	**0**	**0**	**0**	**0**	**0**	**1 (3·4)**	**0**	**0**
**Lymphadenitis**	0	0	0	0	0	0	0	1 (3·4)	0	0
**Neoplasms benign, malignant and unspecified (incl cysts and polyps)**	**0**	**1 (3·3)**	**0**<br>**0**	**0**	**1 (2·6)**	**0**	**0**	**0**	**0**	**0**
**Seborrheic keratosis**	0	0	0	0	1 (2·6)	0	0	0	0	0

Data are n (%)	Study 1 (MOR106/placebo administered by i.v. infusion)						Study 3 (MOR106/placebo administered by s.c. injection)		Study 4 (MOR106/placebo administered by s.c.)	
	**Placebo Q2W (n=37)**	**MOR106 1 mg/kg Q4W (n=30)**	**MOR106 3 mg/kg Q4W (n=30)**	**MOR106 1 mg/kg Q2W (n=36)**	**MOR106 3 mg/kg Q2W (n=38)**	**MOR106 10 mg/kg Q2W (n=36)**	**Placebo Q2W (n=14)**	**MOR106 320 mg Q2W (n=29)**	**Placebo Q2W + TCS OD (n=11)**	**MOR106 320 mg Q2W + TCS OD (n=22)**
Skin papilloma	0	1 (3·3)	0	0	0	0	0	0	0	0
Squamous cell carcinoma	0	0	0	0	0	0	0	0	1 (9·1)	0
**Eye disorders**	**0**	**0**	**0**	**0**	**0**	**1 (2·8)**	**0**	**0**	**0**	**0**
Swelling of eyelid	0	0	0	0	0	1 (2·8)	0	0	0	0
**Gastrointestinal disorders**	**0**	**0**	**0**	**0**	**1 (2·6)**	**0**	**0**	**0**	**0**	**0**
Cheilosis	0	0	0	0	1 (2·6)	0	0	0	0	0
**General disorders and administration site conditions**	**0**	**0**	**0**	**0**	**0**	**1 (2·8)**	**0**	**0**	**0**	**0**
Soft tissue inflammation	0	0	0	0	0	1 (2·8)	0	0	0	0

All studies: data are n (%) for safety population analysis set, from start of treatment to follow up visit.
i.v., intravenous; OD, once-daily; Q2W, every 2 weeks; PT, preferred term; Q4W, every 4 weeks; s.c., subcutaneous; SOC, system organ class; TCS, topical corticosteroid; TEAE, treatment emergent adverse event

Baseline demographics and characteristics, healthy male study participants

Supplementary Table S9: Summary of baseline demographics and characteristics, healthy male study participants

Data are n (%)	Study 2<br>MOR106<br>1 mg/kg s.c.<br>(n=8)	MOR106<br>2 mg/kg s.c.<br>(n=8)	MOR106<br>4 mg/kg Q2W s.c.<br>(n=8)	Total for s.c. doses<br>(n=24)	MOR106<br>4 mg/kg i.v.<br>(n=8)	Total<br>(N=32)
**Age, years**<br>**Median (range)**	32·5 (27–47)	32·0 (23–49)	37·5 (20–50)	33·0 (20–50)	31·5 (23–48)	33·0 (20–50)
**Male, n (%)**	8 (100)	8 (100)	8 (100)	24 (100)	8 (100)	32 (100)
**Race, n (%)**<br>**Asian**	0 (0)	1 (12·5)	1 (12·5)	2 (8·7)	1 (12·5)	3 (9·7)
**White**	7 (100)	7 (87·5)	7 (87·5)	21 (91·3)	7 (87·5)	28 (90·3)
**Weight, kg**<br>**Median (range)**	72·6 (20·1–28·8)	24·0 (21·6–27·4)	25·7 (21·0–29·0)	24·0 (21·0–29·0)	23·5 (20·9–27·1)	24·0 (20·1–29·0)
**BMI, kg/m^2**<br>**Median (range)**	23·5 (20·1–28·8)	24·0 (21·6–27·4)	25·7 (21·0–29·0)	24·0 (20·1–29·0)	23·5 (20·9–27·1)	24·0 (20·1–29·0)

One study participant had missing data for race.

BMI, body mass index; i.v., intravenous; Q2W, every 2 weeks; Q4W, every 4 weeks; s.c., subcutaneous.

PK profile, healthy male study participants

Supplementary Figure S2: Mean MOR106 serum concentration over time with i.v. and s.c. administration of a single dose of MOR106 in healthy male study participants

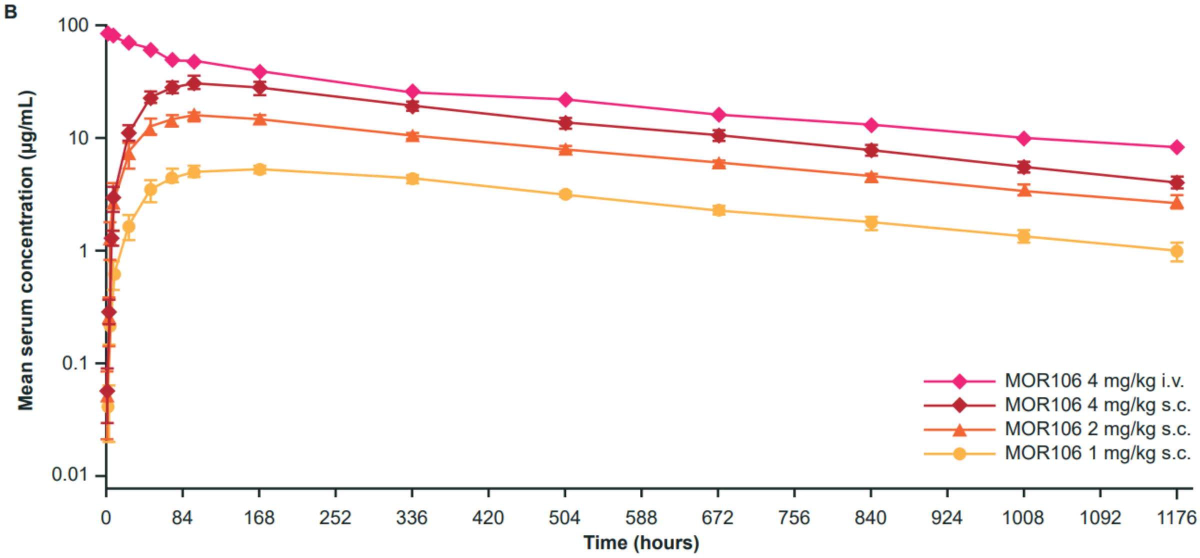

Data are per the PK analysis set. Concentration plotted using a log-linear scale. Error bars show standard error.
i.v., intravenous; s.c., subcutaneous

Supplementary Table S10: Summary of MOR106 PK Parameters, healthy male study participants

	MOR106 1 mg/kg s.c. (N=8)	MOR106 2 mg/kg s.c. (N=8)	MOR106 4 mg/kg s.c. (N=8)	MOR106 4 mg/kg i.v. (N=8)
**C_{max} (µg/mL)**	5·595 (34·93)	16·300 (31·82)	31·925 (23·47)	87·375 (18·54)
**C_{max}/D (µg/mL/mg/kg)**	5·595 (34·93)	8·150 (31·82)	7·981 (23·47)	Not determined
**t_{max} (hour)**	130·745 (94·00–335·62)	94·195 (46·05–169·30)	95·395 (70·78–168·42)	3·110 (2·10–9·12)
**$t_{1/2}$ (day)**	17·21 (26·73)	17·93 (27·67)	15·51 (7·74)	19·04 (16·83)*
**$AUC_{0–t}$ (µg*h/mL)**	3377·9 (30·38)	8933·6 (22·55)	15911·0 (15·11)	25408·8 (23·08)
**$AUC_{0–t}$/D (µg*h/mL/mg/kg)**	3377·9 (30·38)	4466·8 (22·55)	3977·7 (15·11)	Not determined
**$AUC_{0–\infty}$ (µg*h/mL)**	4028 (33·72)	10746 (27·05)	18132·0 (15·63)	32740·9 (12·92)*
**$AUC_{0–\infty}$/D (µg*h/mL/mg/kg)**	4028 (33·72)	5373·4 (27·05)	4533·0 (15·63)	Not determined

Values are mean (%-coefficient of variation), except t_{max} which is median (min–max).
*Value based on seven study participants only.
$AUC_{0–t}$, area under the plasma concentration-time curve from time zero till the last observed quantifiable concentration; $AUC_{0–\infty}$, area under the plasma concentration-time curve from time zero to infinity; C_{max}, maximum observed plasma concentration; i.v., intravenous; s.c., subcutaneous; t_{max}, time of occurrence of C_{max}; D, dose; $T_{1/2}$, terminal elimination half-life

Target binding of IL-17C by MOR106 in patients with moderate–severe AD

Supplementary Figure S3: Serum concentration of total (free and MOR106-bound) IL-17C in study 1

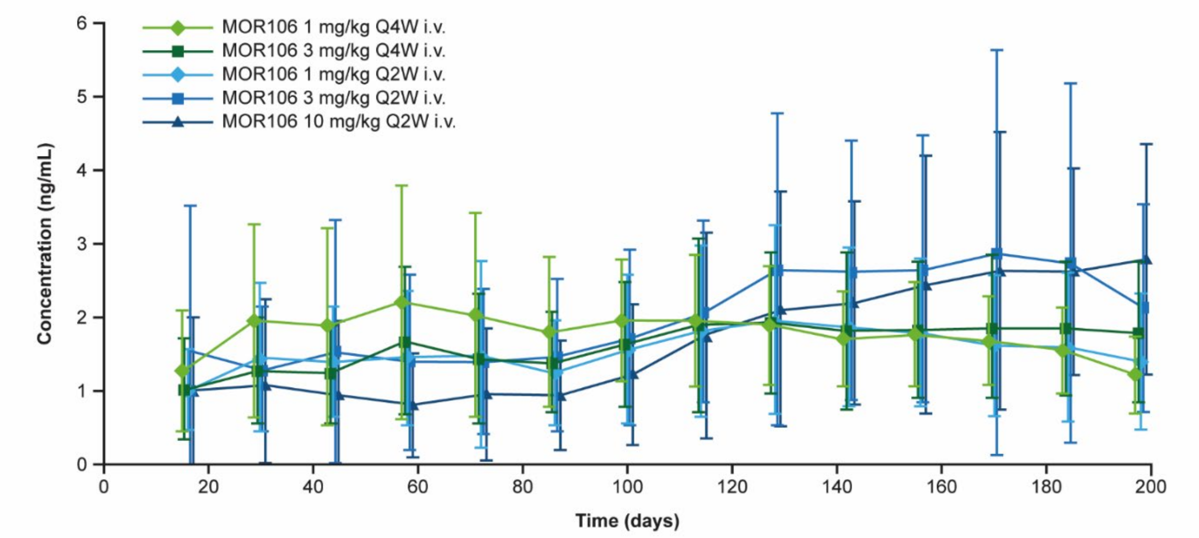

MOR106 administered by i.v. infusion. Data are mean values for the safety analysis set. Error bars show standard deviation of the mean. Binding assay: lower limit of quantification, 80 pg/mL; prior to treatment with MOR106, all values were below the lower limit of quantification.
IL, interleukin; i.v., intravenous; Q2W, every 2 weeks; Q4W, every 4 weeks

Safety, healthy male study participants

Supplementary Table S11: Summary of TEAEs in healthy male study participants

	**Study 2**					
**Data are n (%)**	**MOR106 1 mg/kg s.c. (N=8)**	**MOR106 2 mg/kg s.c. (N=8)**	**MOR106 4 mg/kg s.c. (N=8)**	**Total for s.c. doses (N=24)**	**MOR106 4 mg/kg i.v. (N=8)**	**Total (N=32)**
**TEAE**	7 (87·5)	4 (50)	7 (87·5)	18 (75)	5 (62·5)	23 (71·9)
**Serious TEAE**	0	0	0	0	0	0
**Death**	0	0	0	0	0	0
**Infusion-related reaction**	0	0	0	0	0	0
**Injection site-related reaction**	0	0	1 (12·5)	1 (4·2)	0	1 (3·1)
**Skin related TEAE**	4 (50)	2 (25)	3 (37·5)	9 (37·5)	3 (37·5)	12 (37·5)
**Worst TEAE intensity***						
**Mild**	6 (75)	4 (50)	5 (62·5)	15 (62·5)	5 (62·5)	20 (62·5)
**Moderate**	1 (12·5)	0	2 (25)	3 (12·5)	0	3 (9·4)
**Drug-related TEAE**†	4 (50)	1 (12·5)	4 (50)	9 (37·5)	4 (50)	13 (40·6)
**Discontinued**	0	0	0	0	0	0

*No severe or life-threatening TEAEs were reported.
†As determined by the investigator, or missing drug relatedness information.
Data are per the safety analysis set.
i.v., intravenous; s.c., subcutaneous; TEAEs, treatment-emergent adverse events.

Supplementary Table S12: Summary of TEAEs by SOC and PT in healthy male study participants occurring in ≥2 individuals

Data are n (%)	Study 2<br>MOR106<br>1 mg/kg s.c.<br>(N=8)	MOR106<br>2 mg/kg s.c.<br>(N=8)	MOR106<br>4 mg/kg s.c.<br>(N=8)	MOR106<br>4 mg/kg i.v.<br>(N=8)	Total<br>(N=32)
**Any TEAE**	7 (87·5)	4 (50)	7 (87·5)	5 (62·5)	23 (71·9)
**Infections and infestations**					
**Rhinitis**	2 (25)	0	2 (25)	1 (12·5)	5 (15·6)
**Nasopharyngitis**	0	1 (12·5)	2 (25)	0	3 (9·4)
**Tinea versicolor**	1 (12·5)	0	1 (12·5)	0	2 (6·3)
**Skin and subcutaneous tissue disorders**					
**Dermatitis acneiform**	2 (25·0)	1 (12·5)	1 (12·5)	3 (37·5)	7 (21·9)
**Nervous system disorders**					
**Headache**	2 (25·0)	1 (12·5)	0	0	3 (9·4)
**Dizziness**	1 (12·5)	0	1 (12·5)	0	2 (6·3)
**Respiratory, thoracic and mediastinal disorders**					
**Cough**	0	0	1 (12·5)	1 (12·5)	2 (6·3)
**Oropharyngeal pain**	2 (25·0)	0	0	0	2 (6·3)
**General disorders and administration site conditions**					
**Medical device site rash**	0	1 (12·5)	0	1 (12·5)	2 (6·3)

Data are per the safety analysis set.
i.v., intravenous; PT, preferred term; s.c., subcutaneous; SOC, system organ class; TEAEs, treatment-emergent adverse events.

Institutional Review Board Statement

Study protocols were approved by the appropriate ethics committee/institutional review board at each participating study site (for further details see Supplementary Table S13).

Supplementary Table S13: Ethics committee approval information

Ethics committee / institutional review board name	Study sites	Date of initial approval	EC / IRB approval code
**Study 1 (MOR106-CL-201)**			
East Midlands – Leicester Central, Leicester, United Kingdom	GBR001,<br>GBR002,<br>GBR003,<br>GBR004	09 May 18<br>02 Nov 18<br>02 Nov 18<br>09 May 18	18/EM/0113<br>18/EM/0113<br>18/EM/0113<br>18/EM/0113
Etická komisia Univerzitná nemocnica, Bratislava, Slovakia	SVK 001 (and Central EC)	15 Apr 19	135/95
Komisja Bioetyczna Przy Dolnośląskiej Izbie Lekarskiej, Wrocławiu, Poland	POL002,<br>POL003,<br>POL004,<br>POL005,<br>POL006,<br>POL007,<br>POL009,<br>POL011,<br>POL012,<br>POL013,<br>POL014,<br>POL015,<br>POL016,<br>POL017,<br>POL018,<br>POL019,	13 Jun 18<br>13 Jun 18<br>13 Jun 18<br>13 Jun 18<br>13 Jun 18<br>13 Jun 18<br>13 Jun 18<br>13 Jun 18<br>13 Jun 18<br>13 Jun 18<br>13 Jun 18<br>13 Jun 18<br>13 Jun 18<br>13 Jun 18<br>13 Jun 18<br>16 Jan 19	N° 16/06/2018<br>N° 16/06/2018<br>N° 16/06/2018<br>N° 16/06/2018<br>N° 16/06/2018<br>N° 16/06/2018<br>N° 16/06/2018<br>N° 16/06/2018<br>N° 16/06/2018<br>N° 16/06/2018<br>N° 16/06/2018<br>N° 16/06/2018<br>N° 16/06/2018<br>N° 16/06/2018<br>N° 16/06/2018<br>N° 03/Op/19

	POL021,<br>POL022,<br>POL023,<br>POL024,<br>POL025,<br>POL026,<br>POL027,<br>POL028,<br>POL029,<br>POL030,<br>POL031	12 Dec 18<br>12 Dec 18<br>12 Dec 18<br>12 Dec 18<br>12 Dec 18<br>12 Dec 18<br>16 Jan 19<br>10 Apr 19<br>10 Apr 19<br>10 Apr 19<br>10 Apr 19	N° 373/Op/2018<br>N° 373/Op/2018<br>N° 373/Op/2018<br>N° 373/Op/2018<br>N° 373/Op/2018<br>N° 373/Op/2018<br>N° 03/Op/19<br>N° 90/2019<br>N° 90/2019<br>N° 90/2019<br>N° 90/2019
Ethik-kommission der Universität, Lübeck, Germany	DEU001,<br>Central EC for the Study in Germany	02 Feb 18	17-375
Ethikkommission an der Medizinischen Fakultät der Rheinischen Friedrich- Wilhelms-Universität, Bonn, Germany	DEU002	02 Feb 18	17-375
Ethikkommission der Fakultät für Medizin der Technischen Universität München, Munich, Germany	DEU003	02 Feb 18	17-375
Ethik-Kommission der Ärztekammer Westfalen-Lippe und der Westfälischen Wilhelms-Universität, Munich, Germany	DEU004,<br>DEU017,<br>DEU019	02 Feb 18<br>21 Jun18<br>20 Apr 18	17-375<br>17-375<br>17-375

Ethik-Kommission bei der Landesärztekammer Baden-Württemberg, Stuttgart, Germany	DEU005,<br>DEU022	20 Apr 18<br>20 Sep 19	17-375<br>17-375
Ethik-Kommission bei der Landesärztekammer Rheinland-Pfalz, Mainz, Germany	DEU007	20 Apr 18	17-375
Ethikkommission bei der Ärztekammer Niedersachsen, Hannover, Germany	DEU008,<br>DEU014	02 Feb 18<br>28 May 18	17-375<br>17-375
Ethik-Kommission der Ärztekammer Hamburg - Körperschaft des öffentlichen Rechts, Hamburg, Germany	DEU010	02 Feb 18	17-375
Ethikkommission bei der LMU München, Munich, Germany	DEU011	20 Apr 18	17-375
Landesamt für Gesundheit und Soziales Berlin – Geschäftsstelle der Ethik-Kommission, Berlin, Germany	DEU012,<br>DEU023	20 Apr 18<br>28 Nov 18	17-375<br>17-375
Ethik-Kommission der Landesärztekammer Brandenburg Geschäftsstelle, Cottbus, Germany	DEU013	20 Apr 18	17-375

Ethik-Kommission des Fachbereichs Medizin Universitätsklinikum der Goethe-Universität, Frankfurt, Germany	DEU015	20 Apr 18	17-375
Ethik-Kommission der Medizinischen Fakultät der Ruhr-Universität Bochum, Bochum, Germany	DEU018	27 Jun18	17-375
Ethikkommission der Medizinischen Fakultät Heidelberg, Heidelberg, Germany	DEU021	02 Aug 18	17-375
Egészségügyi Tudományos Tanács Klinikai Farmakológia Etikai Bizottsága, Budapest, Hungary	HUN001, HUN002, HUN003, HUN004, HUN005	26 Feb 18 - All sites	5342-0/2018-EKL
**Studies 2 and 3 (MOR106-CL-102 part 1 / part 2)**			
Ethik-Kommission der Medizinischen Fakultät der FAU Erlangen-Nürnberg, Erlangen, Germany	DEU005	19 Dec 18	2018-482-f-A
Ethikkommission an der Technischen Universität Dresden, Dresden, Germany	DEU003	19 Dec 18	2018-482-f-A

Ethikkommission<br>Otto-von- Guericke-Universität,<br>Magdeburg, Germany	DEU007	19 Dec 18	2018-482-f-A
South Central – Berkshire B Research Ethics Committee, Bristol, United Kingdom	GBR001,<br>GBR003,<br>GBR004,<br>GBR005	28 Jun 18<br>24 Jun 19<br>24 Jun 19<br>24 Jun 19	18/SC/0246<br>18/SC/0246<br>18/SC/0246<br>18/SC/0246
CEIm Hospital Clinic de Barcelona, Barcelona, Spain	ESP003,<br>ESP005,<br>ESP006	28 Nov 18<br>28 Nov 18<br>28 Nov 18	21/2018<br>21/2018<br>21/2018
Ethics Committee at Medical Center of Limited Liability Company “Medical Center Named by Academician Yuriy Spizhenko”, Kyiv, Ukraine	UKR001	04 Oct 18	Exit MM N°3 - 04 Oct 18
**Study 4 (MOR106-CL-204)**			
Advarra IRB, Columbia, United States of America	All U.S. sites	21 Dec 18	No code or reference recorded on the approval letter
Health Research Ethics Board, NL, Canada	Dr. Wayne Gulliver,<br>NewLab Clinical Research Inc.,<br>187 Lemarchant Rd,<br>St. John’s,<br>Canada,<br>A1C 2H5	06 Mar 19	No code or reference recorded on the approval letter

References

1 ClinicalTrials.gov. NCT03568071. A study to assess efficacy, safety, tolerability and pharmacokinetics (PK)/pharmacodynamics (PD) of MOR106 in subjects with moderate to sever atopic dermatitis (IGUANA). Last update posted 18 March 2020. Available at: https://clinicaltrials.gov/ct2/show/NCT03568071?term=NCT03568071&draw=2&rank=1 (last accessed January 2022).

2 ClinicalTrials.gov. NCT03689829. A study to test safety, tolerability, and the way the body absorbs, distributes, and gets rid of a study drug called MOR106, in healthy subjects and in patients with moderate to severe atopic dermatitis. Last update posted 18 March 2020. Available at: https://clinicaltrials.gov/ct2/show/NCT03689829?term=NCT03689829&draw=2&rank=1 (last accessed January 2022).

3 ClinicalTrials.gov. NCT03864627. A study to evaluate the safety and tolerability of MOR106 administered concomitantly with topical corticosteroids, in adult participants with moderate to severe atopic dermatitis (GECKO). Last update posted 5 January 2021. Available at: https://clinicaltrials.gov/ct2/show/NCT03864627?term=NCT03864627&draw=2&rank=1 (last accessed January 2022).

4 Hanifin JR, G. Diagnostic features of atopic dermatitis. *Acta Derm Venereol Suppl (Stockh)* 1980; **92**: 44-7.

5 Hanifin JM, Thurston M, Omoto M *et al.* The eczema area and severity index (EASI): assessment of reliability in atopic dermatitis. EASI Evaluator Group. *Exp Dermatol* 2001; **10**: 11-8.

6 Rehal B, Armstrong AW. Health outcome measures in atopic dermatitis: a systematic review of trends in disease severity and quality-of-life instruments 1985-2010. *PLoS One* 2011; **6**: e17520.

7 Thaçi D, Constantin M, Rojkovich B *et al.* MOR106, an anti-IL-17C mAb, a potential new approach for treatment of moderate-to-severe atopic dermatitis: Phase 1 study. Abstract presented at the American Academy of Dermatology Annual Meeting. 16–20 February 2018, San Diego, CA. 2018.
